# Novel immune scoring dynamic nomograms based on B7-H3, B7-H4, and HHLA2: Potential prediction in survival and immunotherapeutic efficacy for gallbladder cancer

**DOI:** 10.3389/fimmu.2022.984172

**Published:** 2022-09-08

**Authors:** Chao Lv, Shukun Han, Baokang Wu, Zhiyun Liang, Yang Li, Yizhou Zhang, Qi Lang, Chongli Zhong, Lei Fu, Yang Yu, Feng Xu, Yu Tian

**Affiliations:** ^1^ Department of General Surgery, Shengjing Hospital of China Medical University, Liaoning, China; ^2^ Department of Surgery, Jinzhou Medical University, Liaoning, China

**Keywords:** B7-H3 (CD276), B7-H4 (B7x/B7S1), HHLA2 (B7H7/B7-H5), tumor infiltrating lymphocytes (TILs), gallbladder cancer

## Abstract

**Background:**

Gallbladder cancer (GBC) is a mortal malignancy with limited therapeutic strategies. We aimed to develop novel immune scoring systems focusing on B7-H3, B7-H4, and HHLA2. We further investigated their potential clinical effects in predicting survival and immunotherapeutic efficacy for GBC.

**Methods:**

This was a retrospective cohort study in a single center that explored the expression characteristics of B7-H3, B7-H4, and HHLA2. The immune scoring nomograms for prognostic were developed *via* logistic regression analyses. Their performance was evaluated using the Harrell concordance index (C-index) and decision curves analysis (DCA), and validated with calibration curves.

**Results:**

B7-H3, B7-H4, and HHLA2 manifested with a relatively high rate of co-expression patterns in GBC tissues. They were associated with worse clinicopathological stage, suppression of immune microenvironment, and unfavorable prognosis in postoperative survival. B7 stratification established based on B7-H3, B7-H4, and HHLA2 was an independent prognostic predictor (p<0.05 in both groups). Moreover, immune stratification was also successfully constructed based on B7 stratification and the density of CD8^+^ TILs (all p<0.001). The prediction models were developed based on B7-/or immune stratification combined with the TNM/or Nevin staging system. These novel models have excellent discrimination ability in predicting survival and immunotherapeutic efficacy for GBC patients by DCA and clinical impact plots. Finally, dynamic nomograms were developed for the most promising clinical prediction models (B7-TNM model and Immune-TNM model) to facilitate prediction.

**Conclusions:**

Immune scoring systems focusing on B7-H3, B7-H4, and HHLA2 may effectively stratify the prognosis of GBC. Prognostic nomograms based on novel immune scoring systems may potentially predict survival and immunotherapeutic efficacy in GBC. Further valid verification is necessary.

## Introduction

Gallbladder cancer (GBC) is the most common malignancy in the biliary tract, usually with a poor prognosis (1). Although with a low incidence rate, the mortality rate of GBC is relatively high (2). The curative strategy is limited to surgical resection, but fewer than 10% of patients are eligible (3), and most patients are complicated with unresectable or metastatic GBC (4, 5). Currently, gemcitabine and cisplatin are the main chemotherapeutic regimens for recurrent or metastatic GBC, but they reveal limited therapeutic effects (6, 7). New therapeutic schedules focusing on immunomodulatory drugs have been promising in recent years, other than directly cytotoxic cancer therapies.

The investigation of immunotherapies targeting the tumor microenvironment (TME) is a popular topic. Tumor cells can evade immune surveillance *via* inhibitory checkpoint proteins, which promote T-cell exhaustion with a reduced functional capacity. Immune checkpoint blockade of the PD-1/PD-L1 axis has been beneficial in many advanced solid malignancies with PD-L1 overexpression (8). It has opened a new era in the therapeutic strategies for solid tumors. However, the therapeutic effects are controversial for GBC patients when targeting inhibition of the PD-1/PD-L1 axis. First, a significant proportion of GBC patients could not benefit from this treatment strategy since only 12% to 23% of GBC tissues showed PD-L1 overexpression, according to recent studies (9–12). Several studies recognized PD-L1 as an independent adverse prognostic marker in GBC (11–13), but there are still controversies (10). Second, mismatch repair (MMR) protein is an indicator predicting the response of solid tumors to PD-1 blockade; however, only 1.3% of GBCs showed MMR deficiency (14, 15). Additionally, the frequency of MMR deficiency-induced microsatellite instability (MSI) among Western-world GBC was rare, with a proportion less than 2% (4). Obviously, most GBC patients cannot benefit from anti-PD-1 therapy. Third, there is still no immune scoring system to stratify the prognosis and guide immunotherapy for GBC based on PD-L1 expression and tumor-infiltrating lymphocytes (TILs), most notably due to the low rate of PD-L1 overexpression. Therefore, exploring additional immune checkpoint markers and developing adequate immune classifications or scoring algorithms for GBC is critical.

The B7-CD28 family is phylogenetically divided into three groups, and the third group consists of the newly identified immune checkpoints [B7-H3 (CD276), B7-H4 (B7x/B7S1), and HHLA2 (B7H7/B7-H5)/TMIGD2 (IGPR-1/CD28H)/KIR3DL3], which play essential roles in the peripheral immune regulation (16–18). B7-H3, B7-H4, and HHLA2 (abbreviated as the B7-third group) are type I transmembrane proteins that share varying degrees of identity with PD-L1 (16, 19–21). B7-H3-rich tumors are depleted in CD8^+^ T cells (22, 23). High expression of B7-H3 is associated with a lower level of TILs and poor prognosis (24, 25). However, an inconsistent result from another study showed increased TILs and CD8^+^ T cells within tumors with high B7-H3 expression (26). The precise function of B7-H3 has not been fully recognized since its receptors remain unknown (27). Moreover, the coinhibitory or costimulatory roles of B7-H3 in the immune response (28, 29) may vary in different TMEs. B7-H4 acts as a negative regulator of T cells, promoting tumors to evade immune surveillance by suppressing CD8^+^ TILs in cytotoxic T lymphocytes (CTLs) and infiltrative density (30). However, different tumors revealed inconsistent prognoses (31). Until now, only one study reported that the positive rates of B7-H3 and B7-H4 were 66.67% and 69.0% in GBC tissues, respectively. Both were not expressed in chronic cholecystitis tissues (32). Overexpression of B7-H3 and B7-H4 could lead to poor prognostic and clinical parameters, but the association between B7-H4 and the overall survival rate needs to be further explored (32). HHLA2 is expressed in many tumors with a poor prognosis but indicates a favorable prognosis in pancreatic cancer (33). HHLA2 could lead to an inhibitory TME characterized by decreased T cells, CTLs, and an imbalance between regulatory T cells (Tregs) and CTLs (34). HHLA2 was also revealed not to have overlapping expression with PD-L1 (35). This suggests that other immune checkpoints, such as HHLA2, may play a vital role in tumors that do not express PD-L1 or escape PD-1/PD-L1 blockade (16, 36). It is worthwhile to further explore the roles that the B7-third group could play in the immune response.

A previous study established an B7 score based on B7-H3 and HHLA2 expression, which played a significant predictive value for prostate cancer (28). B7-H3 and B7-H4 have co-expression patterns in esophageal cancer, pancreatic cancer, and esophageal squamous cell carcinoma, acting as independent predictors of poor prognoses and valuable prognostic indicators (37–39). The infiltration of CD8^+^ TILs can independently predict a favorable prognosis (28). Forkhead box protein 3 (FOXP3) is a hallmark of the control function of Tregs, which can directly or indirectly suppress T cells and B cells (40). A high CD8^+^/CD3^+^ TIL ratio and a low CD4^+^Foxp3^+^/CD8^+^ ratio were also associated with better prognosis in research on intrahepatic carcinoma (34). Therefore, it is meaningful to investigate the expression status, co-expression patterns, and clinical significance of the B7-third group, as well as the function of TILs in GBC. To date, there are still no relevant immunophenotypic classification or prognostic prediction models for GBC. As a result, we aimed to develop novel immune scoring systems, including B7 stratification (focusing on the B7-third group) and, subsequently, immune stratification (based on B7 stratification and the density of CD8+ TILs), to stratify the prognosis of GBC patients. We also developed several prognostic nomograms based on the immune scoring systems to investigate and validate their potential clinical effects in predicting survival and immunotherapeutic efficacy for GBC. Finally, we developed dynamic nomograms for the most promising clinical prediction models to facilitate the prediction of GBC.

## Materials and methods

### Patients and samples

We conducted a retrospective cohort study of GBC patients who underwent surgical resection at Shengjing Hospital of China Medical University from January 2011 to October 2020. Patients with a clinical and pathological diagnosis of GBC were included. GBC patients who underwent either radical or palliative resection were considered. Patients with preoperative chemotherapy, concurrence of other malignant tumors, or incomplete clinical and pathological data were excluded from this study. A total of 244 GBC patients were available in our hospital. Considering that the formalin-fixed, paraffin-embedded (FFPE) GBC specimens were stored at room temperature varying for 1 to 10 years, there might be inevitable factors influencing the degradation of archival FFPE tissue sections (41). There might be selection bias when dividing the GBC patients into training and testing groups based on the time of admission or surgery. The initially included GBC patients were randomly 1:1 divided into two groups before follow-ups were performed. Finally, we obtained the training group (95 cases) and testing group (103 cases) and excluded the patients who were lost to follow-up. A total of 198 FFPE specimens of GBC were selected from the surgical database. Clinical data were recorded from the electronic medical records, including patient demographics, tumor location, surgical resection, histological grade, TNM stage [according to the eighth edition of the American Joint Committee on Cancer (AJCC) staging system], and Nevin classification (42). The follow-up was completed in October 2021, with a mean of 32 ± 30 (range from 0 to 117) months. Overall survival (OS) was defined as the duration from GBC surgery to death or follow-up deadlines. Cancer-related survival (CRS) was defined as survival duration associated with GBC or follow-up deadlines. This study was approved by the Clinical Research Ethics Committee of Shengjing Hospital of China Medical University, and verbal or written consent was obtained from all enrolled patients (No. 2019PS036K).

### Immunohistochemistry

Briefly, 3 μm thick formalin-fixed paraffin-embedded specimen slides were prepared for immunohistochemical analyses. The sections were deparaffinized and subjected to antigen retrieval, followed by peroxidase blocking with hydrogen peroxide (3%). After blocking with goat serum, sections were incubated with primary antibodies against B7-H3, B7-H4, HHLA2, CD8, and Foxp3 (anti-CD276 antibody: Abcam, ab105922; anti-B7H4 antibody: Abcam, ab252438; anti-HHLA2 antibody: Abcam, ab214327; anti-CD8α antibody: Abcam, ab237710; anti-Foxp3 antibody: Abcam, ab215206) overnight at 4 °C. After washing with phosphate-buffered saline (PBS), sections were incubated with secondary antibody for 1 hour. Visualization with 3,3′-diaminobenzidine (DAB) for 3 minutes. Nuclei were stained with Harris hematoxylin. Sections were dehydrated and then sealed with neutral gel.

### Evaluation of B7-H3, B7-H4, and HHLA2 expression and the density of TILs

All sections were evaluated by two independent investigators blinded to the clinicopathologic data. Discrepant results were resolved after consensus. B7-H3, B7-H4, and HHLA2 immunostaining in GBC cells was assessed by the H-score, which was generated by multiplying the percentage of immunoreactive cells by their associated staining intensity; meanwhile, the staining intensity was quantified as 0, 1, 2, and 3, representing negative, weak, moderate, and strong expression (43). The density of CD8^+^ and Foxp3^+^ TILs in GBC tissues was evaluated based on the average counts of five independent microscopic fields (400×) within the mesenchyme, representing the infiltration of T cells. Qupath (Version: 0.3.2) and ImageJ software were applied to assess the percentage of positively stained cells and/or staining intensity. The X-tile program (44) determined cutoff values for the H-score and infiltrated immune cells. Finally, the optimal cut-off values for the H-score of B7-H3, B7-H4, and HHLA2 were 60, 60, and 90, respectively, calculated using the X-tile program (44) based on the OS of all GBC patients. The calculated cutoff value for differentiating high/low density of CD8^+^ TILs was 95/mm^2^.

### Model building and statistical analysis

Associations among B7-H3, B7-H4, and HHLA2 expression with clinicopathological and other variables were calculated with χ^2^ or Fisher’s exact test. A visualized correlation matrix was established with the package of “corrplot” in R version 4.1.3 (http://www.r-project.org/). Univariate and multivariate analyses were carried out by Cox proportional hazard regression analysis. Kaplan−Meier curves were used to depict OS and CRS at different expression levels of biomarkers; the log-rank test was used for comparisons. IBM SPSS Statistics 26 and GraphPad Prism version 9.0.0 (86) were used to perfrom all the above statistical calculations. The cutoff values of clinicopathological and other parameters, such as age, pathological differentiation, different pathological staging systems, and tumor size, were all calculated with the X-tile program (44) based on the OS of all GBC patients.

Prognostic nomograms were established using variables from multivariable Cox proportional hazards models to select the most significant predictors for CRS. Corresponding packages in R software were applied to perform all the following programs. The Harrell concordance index (C-index) was used to test the accuracy of the novel established prediction models. Its predictive accuracy was also validated by comparing nomogram-predicted versus observed survival probability and depicted by calibration curves (1000 bootstrap resamples) at 1, 3, and 5 years. External validation was performed based on the primary predictive nomogram with the cases from the testing group. The package of “rms” (https://cran.rstudio.com/bin/macosx/contrib/4.1/rms_6.3-0.tgz) was used to perform all these statistics and visualizations. The potential advantages of novel prediction models were evaluated by comparison with different staging systems, with “rcorrp.cens” in the “Hmisc” (https://cran.rstudio.com/bin/macosx/contrib/4.1/Hmisc_4.7-0.tgz) package. Decision curve analysis (DCA) and clinical impact plots (45) were further applied to investigate potential clinical effects of the novel models with the “rmda” (https://cran.rstudio.com/bin/macosx/contrib/4.1/rmda_1.6.tgz) package. Finally, we developed dynamic nomograms based on the most promising prediction models for GBC survival with the “DynNom” (https://cran.rstudio.com/bin/macosx/contrib/4.1/DynNom_5.0.1.tgz) and “rsconnect” (https://cran.rstudio.com/bin/macosx/contrib/4.1/rsconnect_0.8.27.tgz) packages. The established dynamic nomograms are available on the webpage to facilitate prediction.

## Results

### Clinicopathological characteristics of included GBC patients

The significant characteristics of the included GBC patients are summarized in **Supplemental Table 1**. A total of 244 GBC patients available in our hospital were randomly 1:1 divided into two groups before follow-ups were performed. Finally, we obtained the training group (95 cases) and testing group (103 cases), with a total follow-up rate of 81.15%. There were no significant differences in the clinicopathological characteristics or other parameters of GBC patients between the training and testing groups.

The median overall survival (OS) time was 23 (range from 0 to 117) months, and the median cancer-related survival (CRS) time was 22 (range from 2 to 117) months in the training group. The median OS time was 24 (range from 0 to 115) months, and the median CRS time was 23 (range from 2 to 115) months in the testing group. The OS rates at 1, 3, and 5 years were 63.15%, 33.69%, and 21.05%, respectively; the CRS rates at 1, 3, and 5 years were 62.98%, 32.58%, and 19.10%, respectively, in the training group. The OS rates at 1, 3, and 5 years were 63.11%, 32.04%, and 19.42%, respectively; the CRS rates at 1, 3, and 5 years was 65.60%, 31.91%, and 20.21%, respectively, in the testing group.

### B7-H3, B7-H4, and HHLA2 expression in GBC

**Figure 1:**
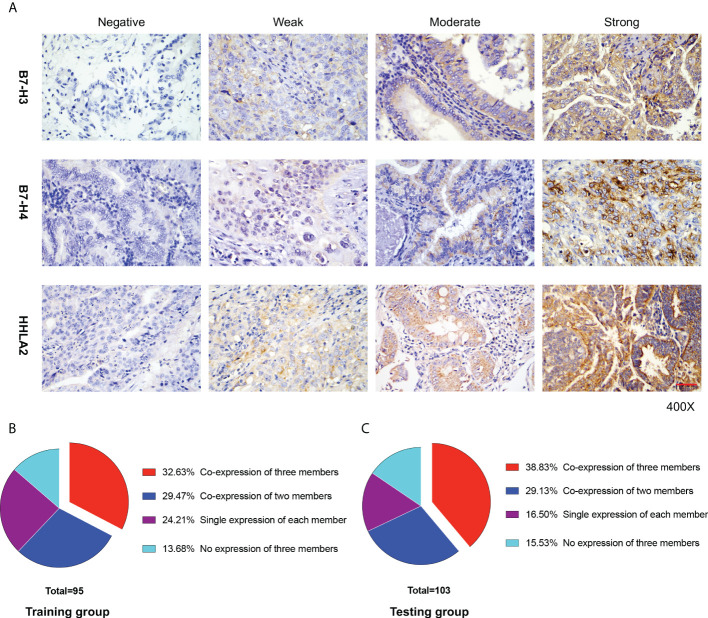
B7-H3, B7-H4, and HHLA2 expression in gallbladder (GBC) tissue. **(A)** Immunohistochemical staining of B7-H3, B7-H4, and HHLA2 in GBC tissues is presented separately. The degree of staining intensity was graded as follows: negative staining, 0; weak staining, 1; moderate staining, 2; and strong staining, 3. Scale bar, 50 μm. **(B, C)** The proportion of GBC patients in different co-expression patterns of the B7-third group (B7-H3, B7-H4, and HHLA2) in the training and testing groups, respectively.

Typical IHC microphotographs of B7-H3, B7-H4, and HHLA2 expression are presented in **Figure 1A**. Correspondingly, high expression of B7-H3, B7-H4, and HHLA2 was identified in 67 (70.53%), 54 (56.84%), and 51 (53.68%) cases in the training group and 70 (67.96%), 70 (67.96%), and 62 (60.19%) cases in the testing group, respectively. Interestingly, acting as the third group of the B7-CD28 immune checkpoint family, B7-H3, B7-H4, and HHLA2 manifested a relatively high rate of co-expression patterns in GBC tissues. (**Figures 1B, C**) The co-expression ratios between B7-H3 and B7-H4, B7-H3 and HHLA2, and B7-H4 and HHLA2 were 46.32%, 43.16%, and 37.89% in the training group and 53.40%, 48.54%, and 43.69% in the testing group, respectively. In addition, the proportion of B7-H3, B7-H3, and HHLA2 co-expression in the training group was 32.63%, while in the validation group, it was 38.83%.

### Relationship between the expression patterns of B7-H3, B7-H4, and HHLA2 and clinicopathological factors in GBC patients

**Figure 2:**
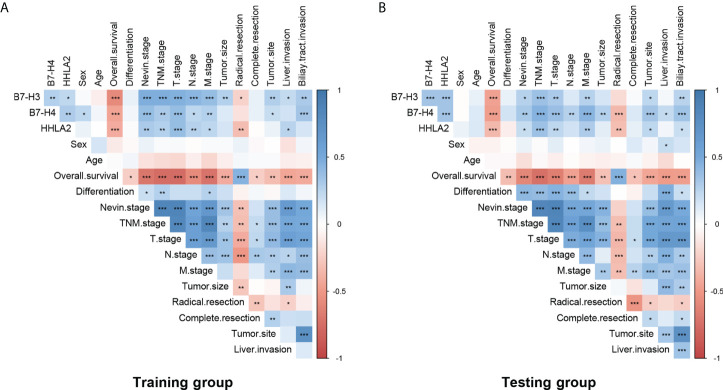
Potential correlation between B7-H3, B7-H4, HHLA2, and clinicopathological factors in patients with GBC. There were significant interrelationships between B7-H3, B7-H4, and HHLA2 in both the **(A)** training group and the **(B)** testing group. Different expression levels (high/low) of B7-H3, B7-H4, and HHLA2 were significantly associated with pathological stages, clinical and surgical parameters, and prognostic survival. (*p < 0.05, **p ≤ 0.01, ***p ≤ 0.001; blank, no significance.).

As listed in **Figure 2** and **Supplemental Table 2**, high expression of B7-H3, B7-H4, and HHLA2 was significantly associated with higher Nevin and TNM stages in both the training and testing groups. The training group showed high expression of B7-H3, B7-H4, and HHLA2 significantly associations with higher T and M stages. However, in the testing group, high expression of B7-H3 was significantly associated with the T stage, and B7-H4 was significantly associated with the T and N stages. All B7-H3, B7-H4, and HHLA2 showed significant associations in the M stage. B7-H3, B7-H4, and HHLA2 were all significantly correlated with T stage in the visual correlation matrix. There were several statistical differences between the visual correlation matrix and **Supplemental Table 2** because the pathological staging system was classified into high/low stages when calculated, but the visual correlation matrix was established based on different stages. Both indicated that high expression of B7-H3, B7-H4, and HHLA2 might predict worse clinicopathological factors in the prognosis of GBC patients.

Meanwhile, high expression of B7-H3, B7-H4 and HHLA2 also showed significant interrelations, both in the Training group (B7-H3 & B7-H4, p=0.007; B7-H3 & HHLA2, p=0.023; B7-H4 & HHLA2, p=0.003) and Testing group (B7-H3 & B7-H4, p=0.001; B7-H3 & HHLA2, p=0.001; B7-H4 & HHLA2, p=0.001). This result indicated that co-expression patterns might exist among the B7-third group.

### Correlation between B7-H3, B7-H4, HHLA2, and TILs in GBC

**Figure 3:**
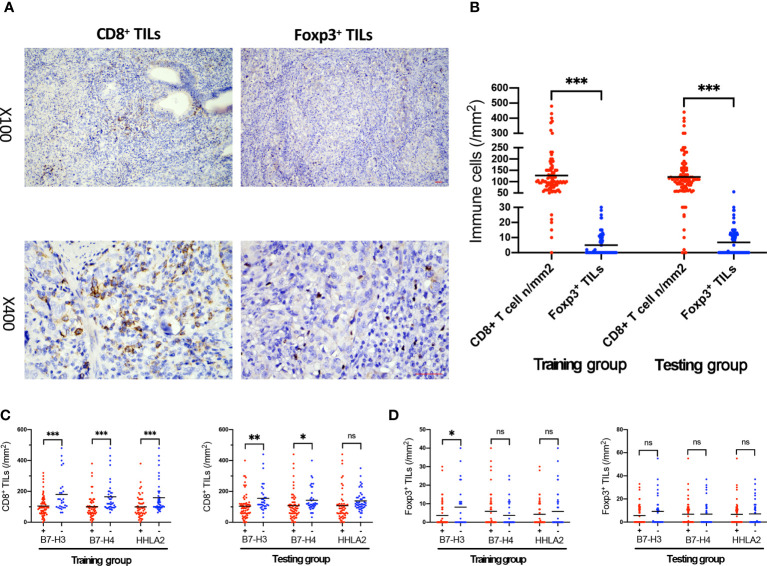
The association of B7-H3, B7-H4, and HHLA2 expression with the density of TILs. **(A)** Immunohistochemical staining of CD8 and Foxp3 expression within tumor tissue; scale bar, 50 μm. **(B)** Scatter plot presenting the density of CD8+ and Foxp3+ TILs in the training and testing groups. **(C)** The density of CD8+ TILs in tumors associated with B7-H3, B7-H4, and HHLA2 expression (+, high expression; -, low expression) in the training and testing groups. **(D)** The density of Foxp3+ TILs in tumors associated with B7-H3, B7-H4, and HHLA2 expression (+, high expression; -, low expression) in the training and testing groups. (Error bars indicate the mean, *p < 0.05, **p ≤ 0.01, ***p ≤ 0.001, ns, no significance.).

A high density of CD8^+^ TILs is associated with improved survival in multiple cancers (46). FOXP3 plays a pivotal role in controlling the function of Tregs, which can directly or indirectly suppress T cells and B cells (40). The expression status of CD8^+^ and Foxp3^+^ TILs deserves to be assessed in GBC tissue. Typical IHC microphotographs of CD8^+^ and Foxp3^+^ TILs are presented in **Figure 3A**. Both training and testing groups showed a much higher density of CD8^+^ TILs than Foxp^3+^ TILs (training group, p<0.001; testing group, p<0.001). High expression of B7-H3, B7-H4, and HHLA2 was associated with a lower density of CD8^+^ TILs in both groups (training group: B7-H3, p<0.001; B7-H4, p=0.001; HHLA2, p=0.001; testing group: B7-H3, p=0.004; B7-H4, p=0.039), except for HHLA2 in the testing group, which showed no significant associations (p=0.066). Meanwhile, the density of Foxp3^+^ TILs was also evaluated between high versus low expression levels of B7-H3, B7-H4, and HHLA2, but no significant differences were identified in either the training and testing groups (training group, B7-H4, p=0.208; HHLA2, p=0.395; testing group, B7-H3, p=0.094; B7-H4, p=0.983; HHLA2, p=0.844), except for B7-H3 in the training group (p=0.017). B7-H3, B7-H4, and HHLA2 might play vital roles in suppressing the immune microenvironment in GBC. (**Figures 3B–D**)

### The prognostic significance of B7-H3, B7-H4, HHLA2, and CD8^+^ TILs in patients with GBC

The univariate analyses for OS and CRS are presented in **Supplemental Table 3**. In both the training and testing groups, high expression of B7-H3, B7-H4, and HHLA2 in GBC tissues was associated with significantly higher risks of unfavorable OS and CRS, but a high density of CD8^+^ TILs was associated with considerably lower risks. Other clinicopathological parameters, including Nevin stage (IV and V), TNM stage (III and IV), tumor size (≥ 3.5 cm), carcinoma located on the neck or cystic duct of the gallbladder, liver or biliary tract invasion, palliative resection, and incomplete resection (namely, rupture) of GBC, were also associated with significantly higher risks of unfavorable OS and CRS.

Correspondingly, the Kaplan−Meier analyses (**Supplemental Figures 1A–D**) showed that GBC patients had significantly shorter OS and CRS with high B7-H3, B7-H4, and HHLA2 expression than GBC patients with low expression. Meanwhile, GBC patients with a high density of CD8^+^ TILs tended to have significantly longer OS and CRS.

Furthermore, multivariate analyses were conducted and are listed in **Supplemental Table 4**. In the training group, high expression of B7-H3, and HHLA2 acted as independent risk factors for unfavorable OS [B7-H3, HR=6.18, 95% CI (2.40 to 15.91), P<0.001; HHLA2, HR=3.67, 95% CI (1.83 to 7.37), P<0.001] and CRS [B7-H3, HR=3.67, 95% CI (1.29 to 10.40), P=0.015; HHLA2, HR=2.64, 95% CI (1.30 to 5.36), P=0.007], and high expression of B7-H4 failed to predict OS [HR=1.34, 95% CI (0.68 to 2.65), P=0.403] and CRS [HR=1.34, 95% CI (0.63 to 2.85), P=0.441]. However, in the testing group, high expression of B7-H3 acted as an independent risk factor both for both unfavorable OS [HR=3.47, 95% CI (1.69 to 7.12), P=0.001] and CRS [HR=4.61, 95% CI (2.1 to 10.12), P<0.001]. High expression of B7-H4 was revealed as an independent risk factor for unfavorable OS [HR=2.58, 95% CI (1.19 to 5.59), P=0.016], and tended to be an independent risk factor for unfavorable CRS; nevertheless, it was not dominant [HR=2.29, 95% CI (0.99 to 5.31), P=0.054]. High expression of HHLA2 failed to independently predict OS [HR=1.24, 95% CI (0.68 to 2.26), P=0.483] or CRS [HR=1.11, 95% CI (0.57 to 2.19), P=0.755]. The high density of CD8^+^ TILs also failed to independently predict OS [training group, HR=0.8, 95% CI (0.41 to 1.56), P=0.515; testing group, HR=0.75, 95% CI (0.46 to 1.25), P=0.271] and CRS [training group, HR=0.53, 95% CI (0.25 to 1.09), P=0.085; testing group, HR=0.71, 95% CI (0.42 to 1.23), P=0.221] in both the training and testing groups, but it might tend to predict a favorable CRS since the p value is less than 0.1 in the training group. The inconsistent results between the training and testing groups might be related to the significant interrelations among B7-H3, B7-H4, and HHLA2. As a result, neither each member of the B7-third group (B7-H3, B7-H4, or HHLA2) nor CD8^+^ TILs could be applied as an independent predictor in the prognosis of GBC.

### The predictive value of B7 stratification based on the B7-third group

A previous study established a new immune scoring system, the B7 score, based on the expression of B7-H3 and HHLA2, which played a vital role in predicting the prognosis of prostate cancer (28). As described in our previous results, acting as the third group of B7-CD28 family members, B7-H3, B7-H4, and HHLA2 had significant interrelations, which might be coexpression patterns. However, B7-H3, B7-H4, and HHLA2 could not be applied as independent predictors in GBC prognosis. We attempted to create a new immune scoring system, B7 stratification, based on the expression of the B7-third group. The GBC patients were divided into four grades of stratification: Grade I, all B7-H3, B7-H4, and HHLA2 had a low level of expression; Grade II, only one variable from B7-H3, B7-H4, and HHLA2 had a high level of expression; Grade III, two variables from B7-H3, B7-H4, and HHLA2 had a high level of expression; and Grade IV, all B7-H3, B7-H4, and HHLA2 had a high level of expression.

**Figure 4:**
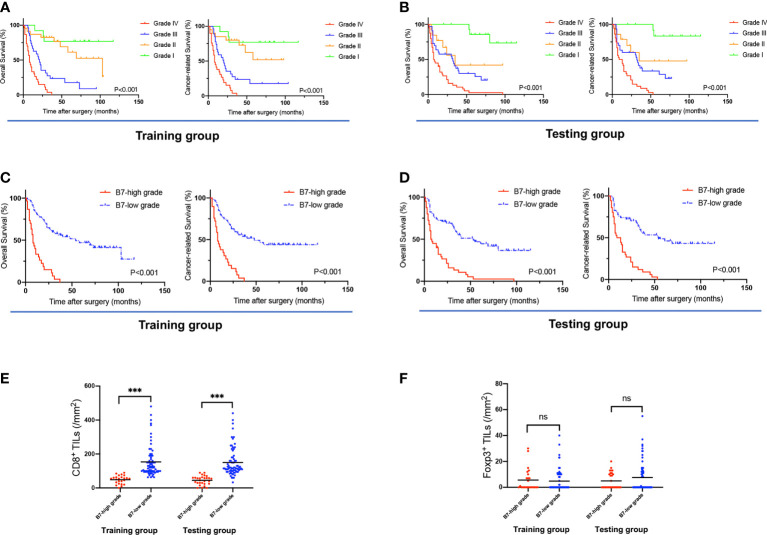
The association of B7 stratification with OS, CRS, and TILs. **(A, B)** Survival curves of OS and CRS, based on B7 stratification, in the training and testing groups. **(C, D)** Survival curves of OS and CRS based on B7-high grade (grade IV of B7 stratification) and B7-low grade (grades I, II, and III of B7 stratification). **(E)** The density of CD8+ TILs in tumors associated with B7 stratification (B7-high/low grade) in the training and testing groups. **(F)** The density of Foxp3+ TILs in tumors associated with B7 stratification (B7-high/low grade) in the training and testing groups. (Error bars indicate the mean, ***p ≤ 0.001, ns, no significance.).

The Kaplan−Meier analyses based on B7 stratification are listed in **Figures 4A, B**. Grade IV presented significantly longer survival than Grades I-III in terms of OS and CRS in both the training and testing groups. However, the training group had no significant differences among Grades I and II in OS or CRS. No significant differences were also revealed between Grades II and III in OS and CRS in the testing group. Therefore, we divided the B7 stratification into two levels: B7-high grade indicates Grade IV of B7 stratification, namely all B7-third group members had high expression; and B7-low grade includes Grades I, II, and III of B7 stratification. This classification was also predicted to be feasible by the X-tile program. As shown in **Supplemental Table 5**, B7-high grade was significantly related to a higher Nevin stage (training group, p<0.001; testing group, p=0.011) and TNM stage (training group, p<0.001; testing group, p=0.006). The Kaplan−Meier analyses based on B7-high/low grades are listed in **Figures 4C, D**. In both the training and testing groups, the B7-high grade group showed a significantly shorter survival duration and higher poor survival rates in terms of OS and CRS than the B7-low grade group (all p<0.001). In the multivariate analyses (**Table 1**), a high grade of B7 stratification was identified as an independent risk factor for predicting unfavorable OS and CRS in both The training [OS, HR=3.51, 95% CI (1.75 to 7.06), P<0.001; CRS, HR=2.36, 95% CI (1.15 to 4.84), P=0.019] and testing groups [OS, HR=2.52, 95% CI (1.37 to 4.62), P=0.003; CRS, HR=2.65, 95% CI (1.30 to 5.41), P=0.007]. A significantly lower density of CD8^+^ TILs was also revealed in the B7-high grade than B7-low grade in both the training and testing groups (all p<0.001). (**Figure 4E**) Meanwhile, there were no significant differences in the density of Foxp3^+^ TILs between the B7-high grade and B7-low grade in both groups (training group, p=0.676; testing group, p=0.253) (**Figure 4F**). This result indicated that B7 stratification based on the B7-third group could successfully predict the prognosis of GBC patients.

**Table 1:** Multivariate analysis of clinicopathological parameters associated with OS and CRS based on B7 stratification.

Variables		Training group								Testing group						
		OS				CRS				OS				CRS		
		HR	95% CI	p		HR	95% CI	p		HR	95% CI	p		HR	95% CI	p
**Differentiation**																
	(Poor, and undifferentiation/	1.34	(0.73 to 2.45)	0.346		1.36	(0.72 to 2.57)	0.350		1.63	(0.91 to 2.95)	0.103		1.53	(0.82 to 2.87)	0.186
	Well, and Moderate)															
**Nevin stage** (IV, V/I, II, III)		8.59	(1.09 to 67.71)	0.041		6.59	(0.81 to 53.44)	0.077		2.43	(0.86 to 6.92)	0.096		2.18	(0.72 to 6.64)	0.17
**TNM stage** (III, IV/I, II)		9.46	(1.38 to 65.09)	0.022		9.68	(1.31 to 71.69)	0.026		1.07	(0.31 to 3.76)	0.915		1.75	(0.47 to 6.54)	0.407
**T stage** (T3, T4/T1, T2)		7.00	(1.97 to 24.83)	0.003		6.45	(1.74 to 24.01)	0.005		1.22	(0.39 to 3.80)	0.737		1.56	(0.51 to 4.78)	0.435
**N stage** (N1, N2/N0)		1.49	(0.60 to 3.69)	0.395		2.07	(0.77 to 5.52)	0.148		1.40	(0.74 to 2.66)	0.3		1.28	(0.68 to 2.39)	0.449
**M stage** (M1/M0)		8.36	(3.79 to 18.42)	<0.001		6.59	(3.04 to 14.29)	<0.001		4.17	(2.00 to 8.70)	<0.001		3.40	(1.56 to 7.38)	0.002
**B7 stratification** (IV/I, II, III)		3.51	(1.75 to 7.06)	<0.001		2.36	(1.15 to 4.84)	0.019		2.52	(1.37 to 4.62)	0.003		2.65	(1.30 to 5.41)	0.007
**CD8** (high/low)		0.77	(0.39 to 1.51)	0.442		0.52	(0.24 to 1.11)	0.089		0.77	(0.47 to 1.25)	0.281		0.71	(0.42 to 1.19)	0.195
**Size** (≥3.5 cm/<3.5 cm)		1.37	(0.72 to 2.63)	0.340		1.40	(0.70 to 2.84)	0.344		1.33	(0.68 to 2.63)	0.408		1.42	(0.65 to 3.11)	0.387
**Tumor site**																
	(Neck, cystic duct/	1.92	(0.85 to 4.33)	0.118		1.85	(0.78 to 4.39)	0.160		1.08	(0.47 to 2.52)	0.85		1.07	(0.44 to 2.59)	0.875
	Fundus, body)															
**Liver invasion** (Yes/No)		2.32	(1.11 to 4.85)	0.025		2.36	(1.04 to 5.34)	0.039		1.59	(0.81 to 3.11)	0.175		1.79	(0.86 to 3.74)	0.123
**Biliary tract invasion** (Yes/No)		1.73	(0.77 to 3.88)	0.183		1.87	(0.75 to 4.66)	0.180		1.64	(0.72 to 3.73)	0.243		1.49	(0.63 to 3.52)	0.363
**Operation**																
	(Palliative resection/	3.57	(1.70 to 7.50)	0.001		5.26	(2.06 to 13.44)	0.001		4.14	(2.00 to 8.56)	<0.001		3.71	(1.70 to 8.09)	0.001
	Radical resection)															
**Complete resection** (No/Yes)		1.38	(0.67 to 2.85)	0.385		1.44	(0.67 to 3.12)	0.349		1.61	(0.77 to 3.37)	0.208		2.23	(0.99 to 5.04)	0.053

+, high expression; -, low expression; HR, hazard ratio; CI, confident interval; p value <0.05 is statistically significant; OS, overall survival; CRS, cancer-related survival.

### Establishment of immune stratification based on B7 stratification and CD8^+^ TILs

CD8^+^ TILs are a well-known factor predicting favorable prognosis (34), which was also confirmed in our previous analyses. Immune stratification was established based on B7 stratification (high grade vs. low grade) and the density of CD8^+^ TILs (high vs. low). Grade I, defined as B7-high grade and low density of CD8^+^ TILs; Grade II, defined as B7-high grade and high density of CD8^+^ TILs; Grade III, defined as B7-low grade and low density of CD8^+^ TILs; Grade IV, defined as B7-low grade and high density of CD8^+^ TILs. The Kaplan−Meier analyses for immune stratification are listed in **Supplemental Figures 2A**, **B**. There were no significant differences between Grade I and Grade II in immune stratification. Both grades led to the worst prognosis in terms of OS and CRS in the training and testing groups. This means that the B7-high grade might play a prominent role in affecting the prognosis rather than the density of CD8^+^ TILs since the function of CD8^+^ TILs might be suppressed by the B7-third group. Both Grades III and IV showed significantly better prognoses in terms of OS and CRS than Grades I and II in both the training and testing groups (all p<0.001). The positive effects of CD8^+^ TILs seemed to be more prominent in GBC patients with a low grade of B7 stratification since Grade IV of immune stratification tended to be associated with the best prognosis than Grade III; however, it was not prominent in the testing group. In conclusion, immune stratification has been proven effective in stratifying the prognosis of GBC. It might be essential to directly predict the clinical prognosis and guide immunotherapy for GBC in the future.

### Potential advantages of novel established prognostic prediction models for GBC survival

**Figure 5:**
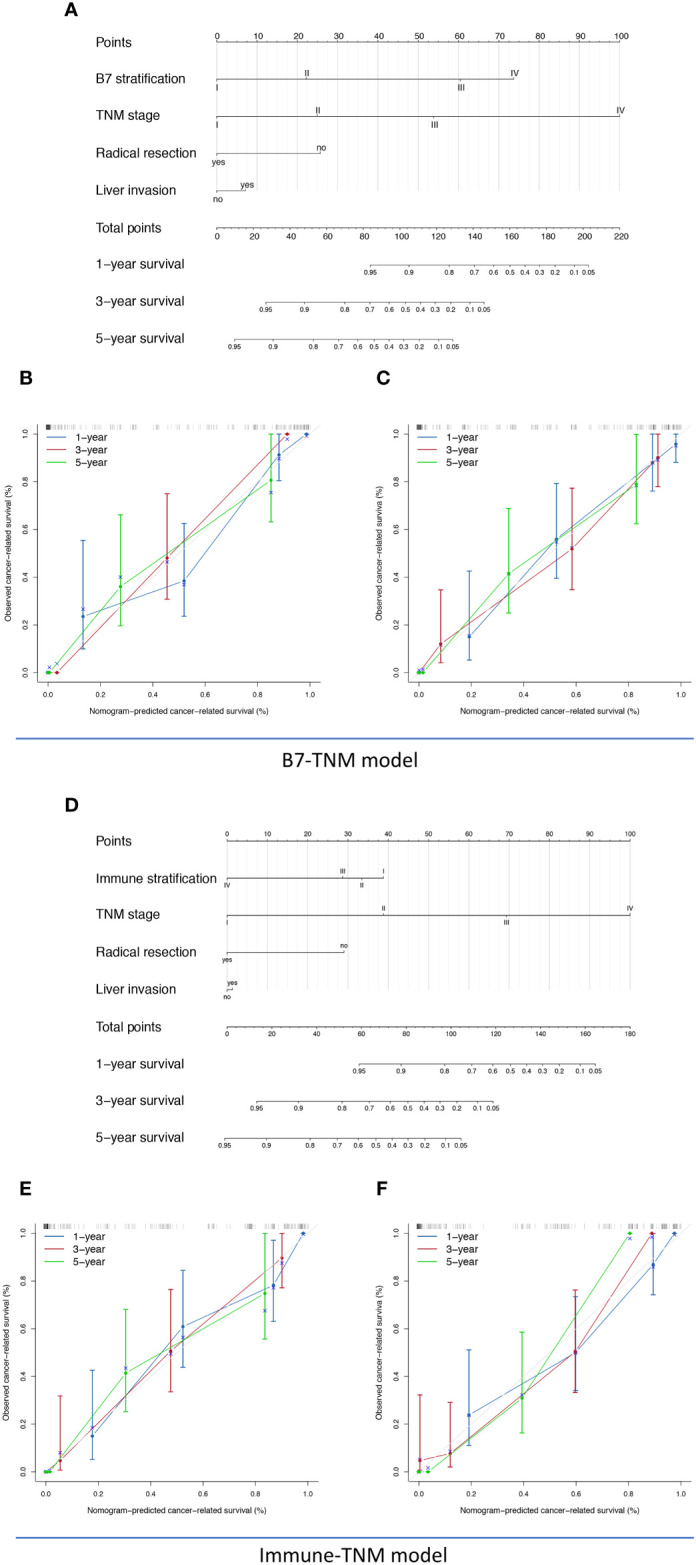
The nomogram, calibration analyses, and external validation of the TNM staging system-based prediction model for cancer-related survival (CRS). **(A)** B7-TNM prediction model established based on TNM stage, B7 stratification, radical resection, and liver invasion; **(B)** The calibration curve of the B7-TNM model in the internal validation; **(C)** The calibration curve of the B7-TNM model in the external validation; **(D)** Immune-TNM prediction model, established based on TNM stage, B7 stratification, radical resection, and liver invasion; **(E)** The calibration curve of the Immune-TNM model in the internal validation; **(F)** The calibration curve of the Immune-TNM model in the external validation .

The prognostic nomograms included all significantly or potentially independent risk factors for CRS of GBC, comprehensively considering the clinical value of each predictor. As previously evaluated, both immune scoring systems, including B7 stratification and immune stratification, took advantage of directly predicting GBC’s clinical prognosis. However, their forecasting performance has yet to be explored. The visualized nomograms were established based on B7 stratification or immune stratification, different pathological staging systems, and associated risk factors. The novel set prediction models are presented in **Figures 5A, D** and **Supplemental Figures 3A, D**, named the B7-TNM model, Immune-TNM model, B7-Nevin model, and Immune-Nevin models, respectively, according to the included predictors. The c-index for each prediction model and pathological staging system is listed in **Supplemental Table 6**. The B7-TNM model and Immune-TNM model were established based on the TNM staging system, showing excellent discrimination ability for GBC survival (C-index was 0.96 and 0.96, respectively.). In the internal set, the calibration curves of both prediction models demonstrated good consistency between the predicted survival and actual observation for 1-, 3-, and 5-year survival rates after surgery. (**Figures 5B, E**) External validations for the discrimination ability of both models were further evaluated using the testing group data. Validation of both models showed appropriate predicting accuracy (C-index was 0.94 and 0.95, respectively.). Details are listed in **Supplemental Table 6**. Both calibration curves of external validations showed an optimal agreement between the predicted and observed probability of 1-, 3-, and 5-year survival after surgery. (**Figures 5C, F**) B7-Nevin and Immune-Nevin models were established based on the Nevin staging system; both models also showed sound performance in predictive accuracy and reliability. (Details are presented in **Supplemental Figure 3**.)

**Figure 6:**
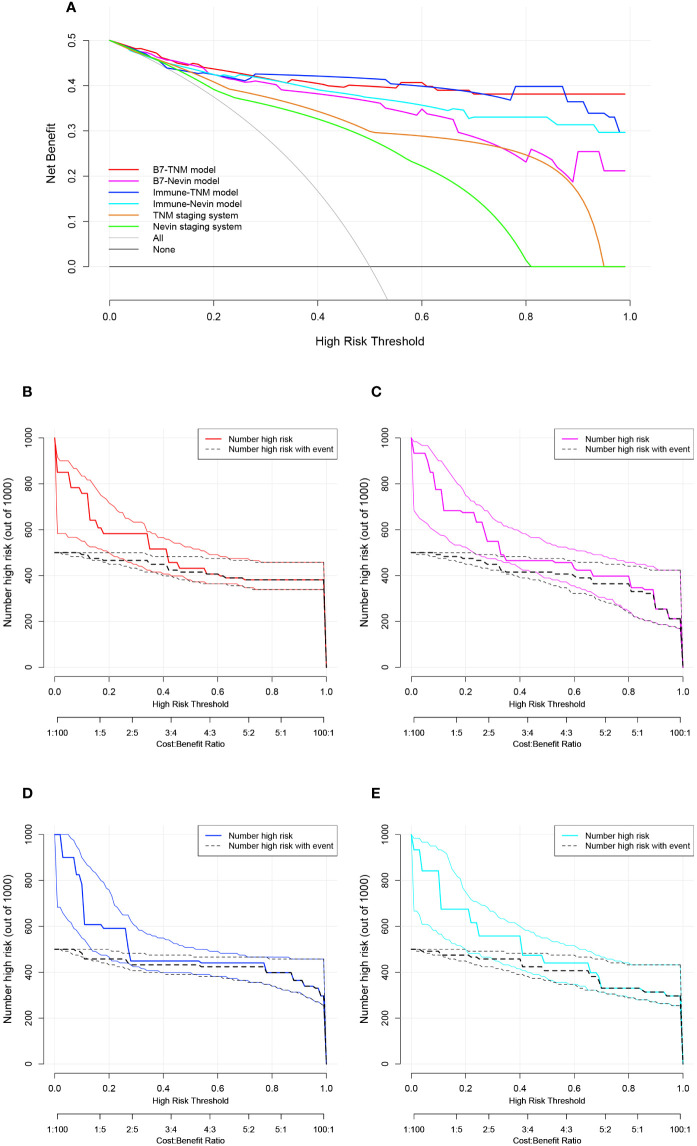
Decision curve analysis (DCA) and clinical impact plots to investigate the potential clinical effects of the novel models. **(A)** The clinical effects were assessed by quantifying the net benefits at different threshold probabilities. A higher net benefit indicated better estimation for decision-making; **(B–E)** Clinical impact curves for the B7-TNM model, B7-Nevin model, Immune-TNM model, and Immune-Nevin model. The two horizontal axes present cost: benefit and corresponding risk threshold. The heavy solid line in different colors for each prediction model shows the estimated high-risk number of 1000 patients, while the black dashed line shows the number of true-positive cases. All plots’ light solid and dashed lines represent the 95% CI constructed by bootstrapping.

Subsequently, the four newly established prediction models were compared with the TNM staging or Nevin staging systems. All models had a trend to display better discrimination ability in predicting GBC survival, although there were no significant statistical differences (0.05 < p value <1). (Details are listed in **Supplemental Table 6**.) DCA was plotted to determine a more delicate and accurate prediction model (**Figure 6A**). Compared with the TNM staging and Nevin staging systems, all the novel established models showed better net benefit across the range of threshold probability for CRS, except for the B7-Nevin model, which crossed with the curve of the TNM staging system. Finally, we identified that the B7-TNM model and Immune-TNM model could be the most recommended prediction models. In contrast, the B7-TNM model had relatively better performance throughout any given threshold. Clinical impact plots were further displayed based on DCA (45) (**Figures 6B–E**). For each novel established model, of 1000 patients, the clinical impact plot showed the estimated number who would have a high risk of GBC-related death for the corresponding risk threshold and visually showed the proportion of those who were true-positive cases. In other words, if the probability of CRS is obtained, we will read out the true-positive risk cases that demand complementary treatment strategies and those that are false-positives.

In conclusion, these results indicated that B7 stratification and immune stratification played a vital role in novel prediction model construction based on pathological staging systems of GBC. B7 stratification/or immune stratification-based nomograms might be potentially powerful predictions for the survival of GBC patients and could be applied with different pathological staging systems to enhance the predictive accuracy of GBC survival. Additionally, dynamic nomograms for the B7-TNM model and Immune-TNM model were developed on the webpage because of their promising performance in the clinical prediction of GBC survival. The B7-TNM prediction model is available on https://dynnomo-for-gallbladder-cancer.shinyapps.io/DynNomapp-B7_TNM_model/, and the Immune-TNM prediction model is available on https://dynnomo-for-gallbladder-cancer.shinyapps.io/DynNomapp-Immune_TNM_model/. Instructions for the B7-TNM prediction model and Immune-TNM prediction model are presented in **Supplemental Figure 4** and **Supplemental Figure 5**, respectively. The multivariate regression parameters for both dynamic models are listed in **Supplemental Table 7**. The novel immune scoring dynamic nomograms will facilitate the prediction of the probability of CRS, which could predict true- or false-positive cases who need corresponding immunotherapeutic strategies and further net benefits.

## Discussion

Immune checkpoint inhibitors have made important achievements in treating malignancies in recent years. The ligands and receptors of the B7-CD28 family play vital roles in T-cell costimulation and coinhibition (28). Immune checkpoint blockade of the PD-1/PD-L1 axis has been proven to be effective in many kinds of tumors (8). However, a low proportion of PD-L1 overexpression (only 12% to 23%) (9–12), MMR protein deficiency (only 1.3%) (14), and MSI (less than 2%) (4) were identified in GBC tissue, all of which indicated that a significant proportion of GBC patients could not benefit from the immunotherapeutic strategies focusing on the PD-L1/PD-1 axis. Further exploring the B7-third group is necessary.

This is the first study to explore the B7-third group in the prognostic prediction of GBC. We found that high expression in the B7-third group was very frequent in GBC tissue. A previous study reported that B7-H3 expression was consistent with B7-H4 in GBC tissue, and a synergetic role might exist between B7-H3 and B7-H4 (32). The simultaneous overexpressing proportion of B7-H3 and HHLA2 was between 18% and 31% in the prostate cancer tissues. Meanwhile, B7-H3 and HHLA2 also showed similar immunological functions and significant associations (28). In our study, we identified a significant proportion of co-expression patterns among the B7-third group in GBC tissues. High expression of B7-H3, B7-H4, or HHLA2 could lead to unfavorable clinicopathological parameters and worse prognoses. The density of CD8^+^ TILs was also significantly reduced when high expression of B7-H3, B7-H4, or HHLA2 occurred. These results indicated that each member of the B7-third group might mediate immune evasion in the TME of GBC. Immunotherapy and prognostic prediction for GBC targeting B7-H3, B7-H4, or HHLA2 might be promising.

We established an B7 stratification based on the frequent co-expression patterns of the B7-third group for GBC. It plays a vital role in stratifying and predicting prognosis. Meanwhile, it might also be valuable in developing precise immunotherapeutic strategies for GBC. For GBC patients with simultaneously high expression of all three members, simultaneous blockade might be an optimal option. The combinatorial blockade should be considered if two of the three members have high expression. However, for patients with high expression of only one member of the B7-third group, a single blockade of the corresponding ligand might be enough.

CD8^+^ TILs have been proven to be of favorable prognostic value in several kinds of tumors, including esophageal cancer, colon cancer, intrahepatic cholangiocarcinoma, and prostate cancer (34, 46, 47). We also found that a high density of CD8^+^ TILs in GBC tissues might benefit a more favorable prognosis, but it was not an independent risk factor. Our further analyses identified that high expression of the B7-third group could lead to a lower density of CD8^+^ TILs, respectively. This result indicated that the B7-third group might influence the prognosis of GBC *via* a suppressive immune microenvironment. Considering the association of CD8^+^ TILs with the prognosis of GBC patients, immune stratification was established further based on B7-high grade/B7-low grade, and high/low density of CD8^+^ TILs. We found that B7-high grade might play a prominent role in affecting the prognosis rather than the density of CD8^+^ TILs. In other words, the positive influence of CD8^+^ TILs on prognosis was relatively weak when all B7-third group members had high co-expression patterns in GBC tissues. This also indicated that the B7-third group might promote the development of GBC beyond the suppression of the immune microenvironment. However, the positive effects of CD8^+^ TILs seemed to be more prominent in GBC patients with a low grade of B7 stratification. As a result, immune stratification has been proven to be effective in stratifying the prognosis of GBC. It might be essential to predict the clinical prognosis and guide immunotherapy for GBC in the future.

Cancer prediction models have become increasingly popular in recent years and play a vital role in personalized interventions for cancers. We had developed several prediction models for GBC survival based on the prognostic prediction ability of B7-/or Immune- stratification and different available pathological staging systems. These novel prediction models also considered clinical and surgical factors, such as the probability of radical resection and liver invasion. We had identified that B7-/or immune stratification-based prediction models played excellent roles in addition to corresponding pathological staging systems. B7-/or immune stratification might enhance the prediction accuracy of different pathological staging systems, although these have already performed relatively well. We further applied DCA to detect the most potent prediction models. The B7-TNM and Immune-TNM models were more promising in accurately predicting GBC survival. They have significant value in clinical settings, providing an accurate early readout of future survival probability in patients with GBC and accurate treatment strategies based on the B7-third group and CD8^+^ TILs. At the same time, these prediction models also considered the clinical progression of GBC and whether a surgical intervention was appropriate. They could be applied to evaluate whether immunotherapy targeting the B7-third group and CD8+TILs is warranted.

B7-H3, an ideal target for cancer immunotherapy, has been widely explored. It was reported that miR-29c overexpression remarkably reduced B7-H3 in ovarian and breast cancer (48, 49). The anti-B7-H3 antibody 8H9 (omburtamab) has shown clinical potential in treating central nervous system malignancies and non-small cell lung cancer, which promoted Fc-dependent NK cells through antibody-dependent cell-mediated cytotoxicity (ADCC) function (50, 51). The adoptive immunotherapy that utilizes effector lymphocytes expressing tumor-specific antibodies based on B7-H3 has been widely explored in different tumors, including chimeric antigen receptors (CARs) (52, 53) and bispecific killer cell engagers (BiKE) (27, 54). Some studies have identified TLT2 as a receptor for B7-H3, which plays a costimulatory role in T-cell activation (29). However, the coinhibitory receptors for B7-H3 on T cells remain unknown. The receptor for H7-H4 also remains to be identified. The current immunotherapeutic strategies based on B7-H4 are still preclinical studies, namely, specific antibody-drug conjugates (ADCs) by binding B7-H4 antibody (55), CAR-T treatment (56), and B7–H4/CD3-bispecific Fab-scFv antibodies (57). For HHLA2, KIR3DL3 is a newly identified coinhibitory receptor on NK cells and T cells that specifically blocks immune inhibitory activity but spares the costimulatory activity of TMIGD2 (17, 35). The KIR3DL3-HHLA2 pathway is a potential immunotherapeutic target for GBC. Overall, the B7-third group is a promising topic targeting immunotherapeutic strategies for GBC. They are worth profoundly exploring.

However, there are several limitations to our study. I) This was a retrospective study, and selection bias may exist. All cases were selected from the same institution in China, and the bias might vary from other institutions or races. II) Since GBC is a relatively rare disease, it took approximately 10 years to obtain these available samples. Considering the inevitable factors that might influence the degradation of archival FFPE tissue sections (41), the GBC patients were randomly 1:1 divided into training and testing groups to avoid bias. However, it might weaken the power of external validation. Moreover, to our knowledge, there are no available public GBC data to further validate the results. There are no recognized cutoff values for the related immune biomarker expression in GBC. They were determined by the X-tile program (44). These discrepancies might exist in other studies. IV) The H-score (43), a semiquantitative analysis, was applied to evaluate the expression level of the B7-third group in GBC. Further investigation on the molecular basis should be performed. V) Survival prediction models were established based on GBC patients only in our hospital. The sample size is limited. A large sample size from multiple centers is necessary to optimize the nomograms and validate their prediction accuracy for GBC. VI) Due to incidental GBC, tumor markers (CEA, CA199, AFP, etc.) were not routinely tested in several patients. These potential survival predictors were not included in our study, which might affect the accuracy of prediction models for GBC survival. VII) The latest nodal (N) category definition has been modified based on the previous seventh edition of the AJCC staging system for GBC (58). Our results from the TNM stage might be different from previous studies.

## Conclusions

This is the first study identifying the co-expression patterns among the B7-third group in GBC tissues. B7 stratification was established based on varied co-expression patterns of the B7-third group, and it could successfully stratify the prognosis of GBC patients. Immune stratification is another prognostic stratification definition depending on the novel B7 stratification and density of CD8+ TILs. This implies a struggle between immunosuppression and immune surveillance. Both stratification strategies independently predicted the prognosis of GBC. Their superior forecasting performance was further proven in the novel developed prediction models by combining with the TNM/or Nevin staging system. Moreover, radical resection and liver invasion are essential issues that should not be ignored. The novel developed prediction models based on B7-/immune stratification might have excellent discrimination ability in predicting GBC survival, especially the B7-TNM and Immune-TNM models. They were meaningful in clinical guidance with an early readout of future survival probability and accurate intervention strategies. Further valid verification is necessary.

## Data availability statement

The original contributions presented in the study are included in the article/**Supplementary Material**. Further inquiries can be directed to the corresponding author.

## Ethics statement

This study was approved by the Clinical Research Ethics Committee of Shengjing Hospital of China Medical University, and verbal or written consent was obtained from all enrolled patients. (No. 2019PS036K). Written informed consent for participation was not required for this study in accordance with the national legislation and the institutional requirements.

## Author contributions

Conceptualization: CL and YT. Data curation: CL, SH, BW, ZL, YL, YZ, QL, CZ, LF and YY. Formal analysis: CL, SH, BW, ZL, YL, YZ, QL, CZ and YT. Funding acquisition. YT. Investigation: CL, SH, BW, ZL, YL, YZ, QL, CZ, LF and YY. Methodology: CL, SH, BW, ZL, YL, YZ, QL, CZ, LF and YT. Project administration: YT. Resources: CL, SH, BW, ZL, YL, YZ, QL, CZ, LF, YY and YT. Software: CL, SH, BW, ZL, YL, YZ, QL and CZ. Supervision: BW and YT. Validation: SH, ZL, YL, YZ, QL, CZ, YY, FX and YT. Visualization: BW, YZ and QL. Writing – original draft: CL. Writing – review & editing: CL, SH, BW, ZL, YL, YZ, QL, CZ, LF, YY, FX and YT. All authors contributed to the article and approved the submitted version.

## Funding

This work was supported in part by the National Natural Science Foundation of China [grant numbers 81974377, 2020], the Scientific Research Project of Education Department of Liaoning Province [grant numbers JC2019017, 2019] and 345 Talent Project [2019–2021].

## Acknowledgments

We would like to thank Jiajun Fu, Wanying Huang from the Department of Pathology, Shengjing Hospital of China Medical University, Yitong Xu from the Department of Pathology, First Hospital of China Medical University, for their assistance in preparing specimens slides and evaluation of IHC staining. Thanks to the company of RStudio for providing “shinyapps.io”, which enables us to link the webpage of our developed prediction models.

## Conflict of interest

The authors declare that the research was conducted in the absence of any commercial or financial relationships that could be construed as a potential conflict of interest.

## Publisher’s note

All claims expressed in this article are solely those of the authors and do not necessarily represent those of their affiliated organizations, or those of the publisher, the editors and the reviewers. Any product that may be evaluated in this article, or claim that may be made by its manufacturer, is not guaranteed or endorsed by the publisher.

## Supplementary material

Supplementary Figure 1 Kaplan−Meier survival curves presenting the associations of B7-H3, B7-H4, and HHLA2 expression, as well as the density of CD8+ TILs, with overall survival (OS) and cancer-related survival (CRS). **(A)** The associations of each parameter with OS in the training group; **(B)** The associations of each parameter with CRS in the training group; **(C)** The associations of each parameter with OS in the testing group; **(D)** The associations of each parameter with CRS in the testing group.

Supplementary Figure 2 Kaplan−Meier survival curves present immune stratification’s association with OS and CRS.

Supplementary Figure 3 The nomogram, calibration analyses, and external validation of the Nevin staging system-based prediction model for cancer-related survival (CRS). **(A)** B7-Nevin prediction model, established based on Nevin stage, B7 stratification, radical resection, and liver invasion; **(B)** The calibration curve of the B7-Nevin model in the internal validation; **(C)** The calibration curve of the B7-Nevin model in the external validation; **(D)** Immune-Nevin prediction model, established based on Nevin stage, B7 stratification, radical resection, and liver invasion; **(E)** The calibration curve of the Immune-Nevin model in the internal validation; **(F)** The calibration curve of the Immune-Nevin model in the external validation.

Supplementary Figure 4 Instructions for the dynamic nomogram of the B7-TNM prediction model on the webpage, Linkage to https://dynnomo-for-gallbladder-cancer.shinyapps.io/DynNomapp-B7_TNM_model/.

Supplementary Figure 5 Instructions for the dynamic nomogram of the Immune-TNM prediction model on the webpage, Linkage to https://dynnomo-for-gallbladder-cancer.shinyapps.io/DynNomapp-Immune_TNM_model/.

## Abbreviations

GBC, gallbladder cancer; TME, tumor microenvironment; B7-third group, including B7-H3, B7-H4, and HHLA2; OS, overall survival; CRS, cancer-related survival; TILs, tumor infiltrating lymphocytes; CTLs, cytotoxic T lymphocytes; FOXP3, Forkhead box protein 3; Treg, regulatory T cells; FFPE, Formalin-fixed, paraffin-embedded; DCA, decision curves analysis; C-index, Harrell concordance index.
