# Supplementary figures and images for: Novel immune scoring dynamic nomograms based on B7-H3, B7-H4, and HHLA2: Potential prediction in survival and immunotherapeutic efficacy for gallbladder cancer
Source: Front Immunol. 2022 Sep 8;13:984172. doi: 10.3389/fimmu.2022.984172 (PMC9493478; doi:10.3389/fimmu.2022.984172)

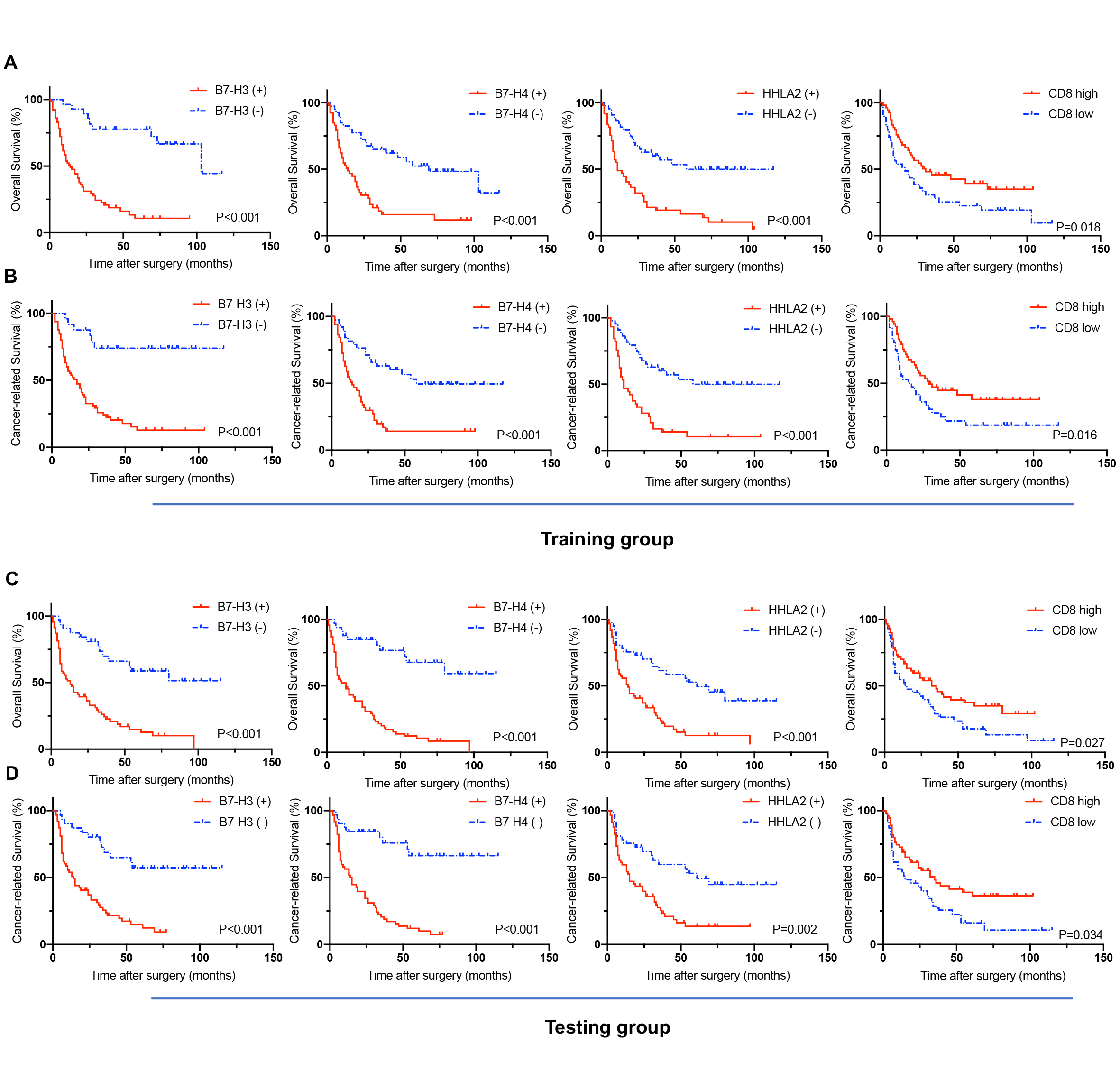

Supplement: Supplementary Figure 1 — Kaplan−Meier survival curves presenting the associations of B7-H3, B7-H4, and HHLA2 expression, as well as the density of CD8+ TILs, with overall survival (OS) and cancer-related survival (CRS). (A) The associations of each parameter with OS in the training group; (B) The associations of each parameter with CRS in the training group; (C) The associations of each parameter with OS in the testing group; (D) The associations of each parameter with CRS in the testing group. [file Image_1.tif]

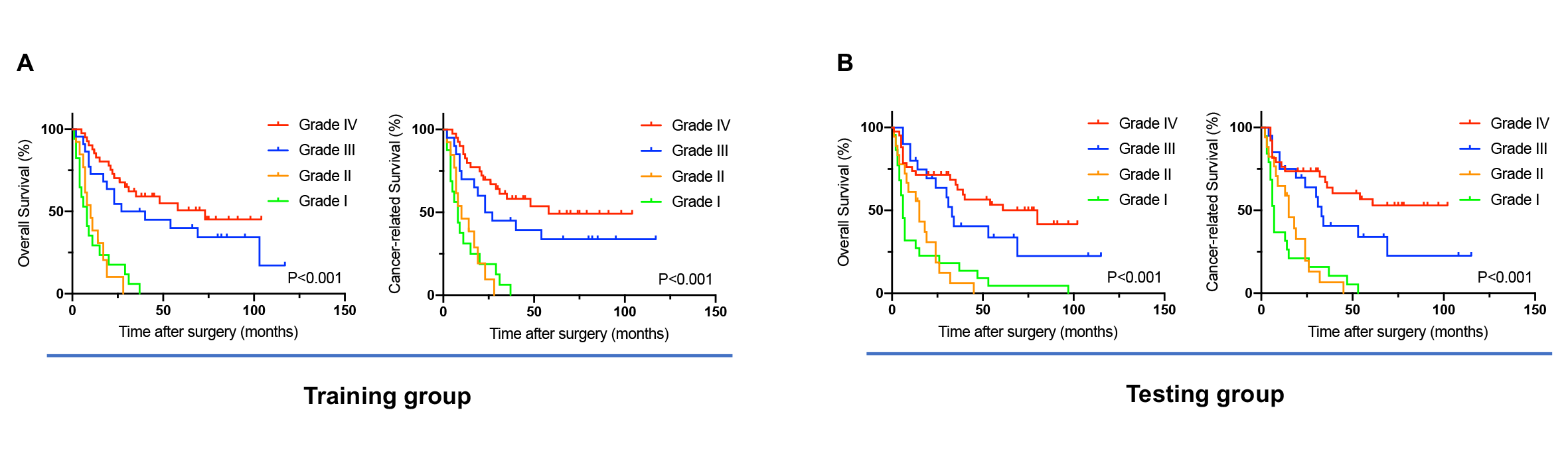

Supplement: Supplementary Figure 2 — Kaplan−Meier survival curves present immune stratification’s association with OS and CRS. [file Image_2.tif]

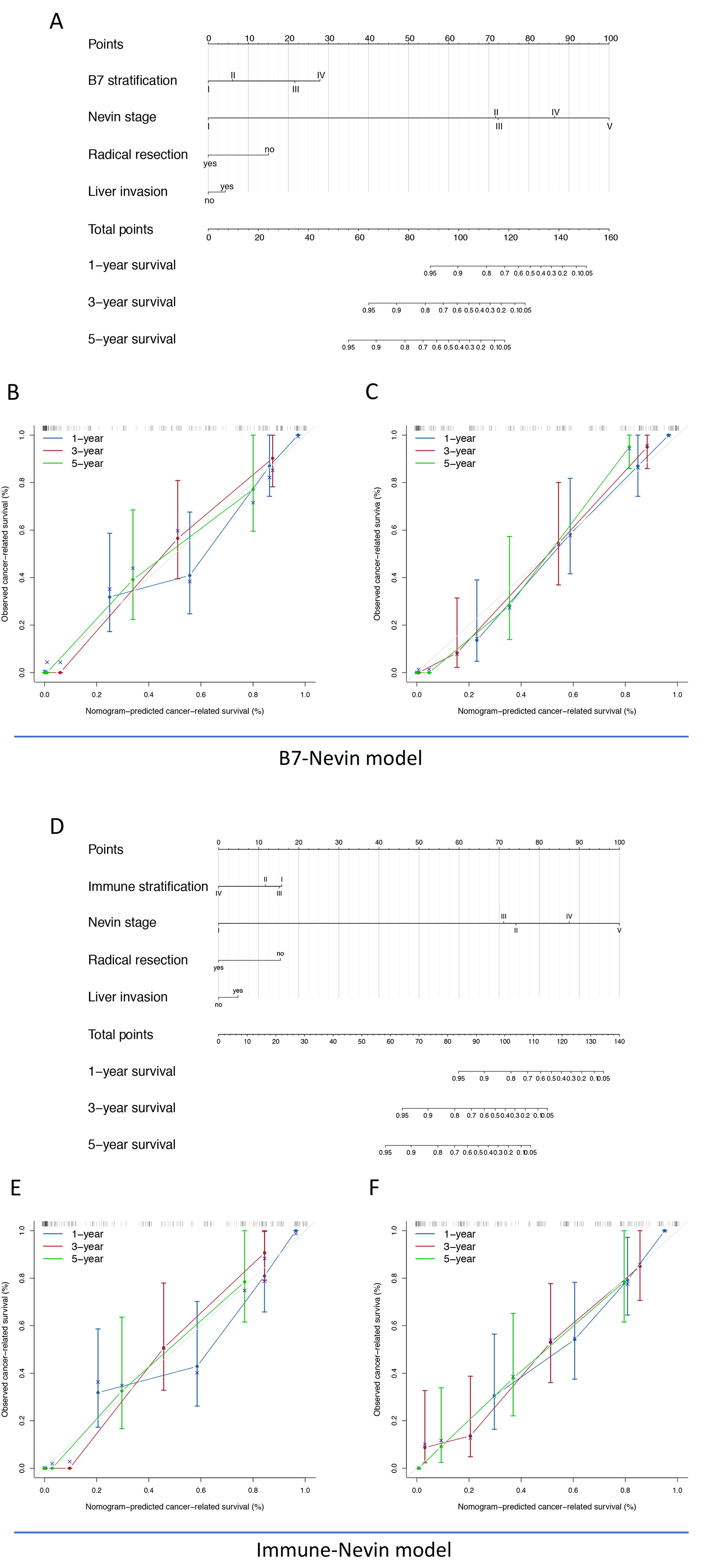

Supplement: Supplementary Figure 3 — The nomogram, calibration analyses, and external validation of the Nevin staging system-based prediction model for cancer-related survival (CRS). (A) B7-Nevin prediction model, established based on Nevin stage, B7 stratification, radical resection, and liver invasion; (B) The calibration curve of the B7-Nevin model in the internal validation; (C) The calibration curve of the B7-Nevin model in the external validation; (D) Immune-Nevin prediction model, established based on Nevin stage, B7 stratification, radical resection, and liver invasion; (E) The calibration curve of the Immune-Nevin model in the internal validation; (F) The calibration curve of the Immune-Nevin model in the external validation. [file Image_3.tif]

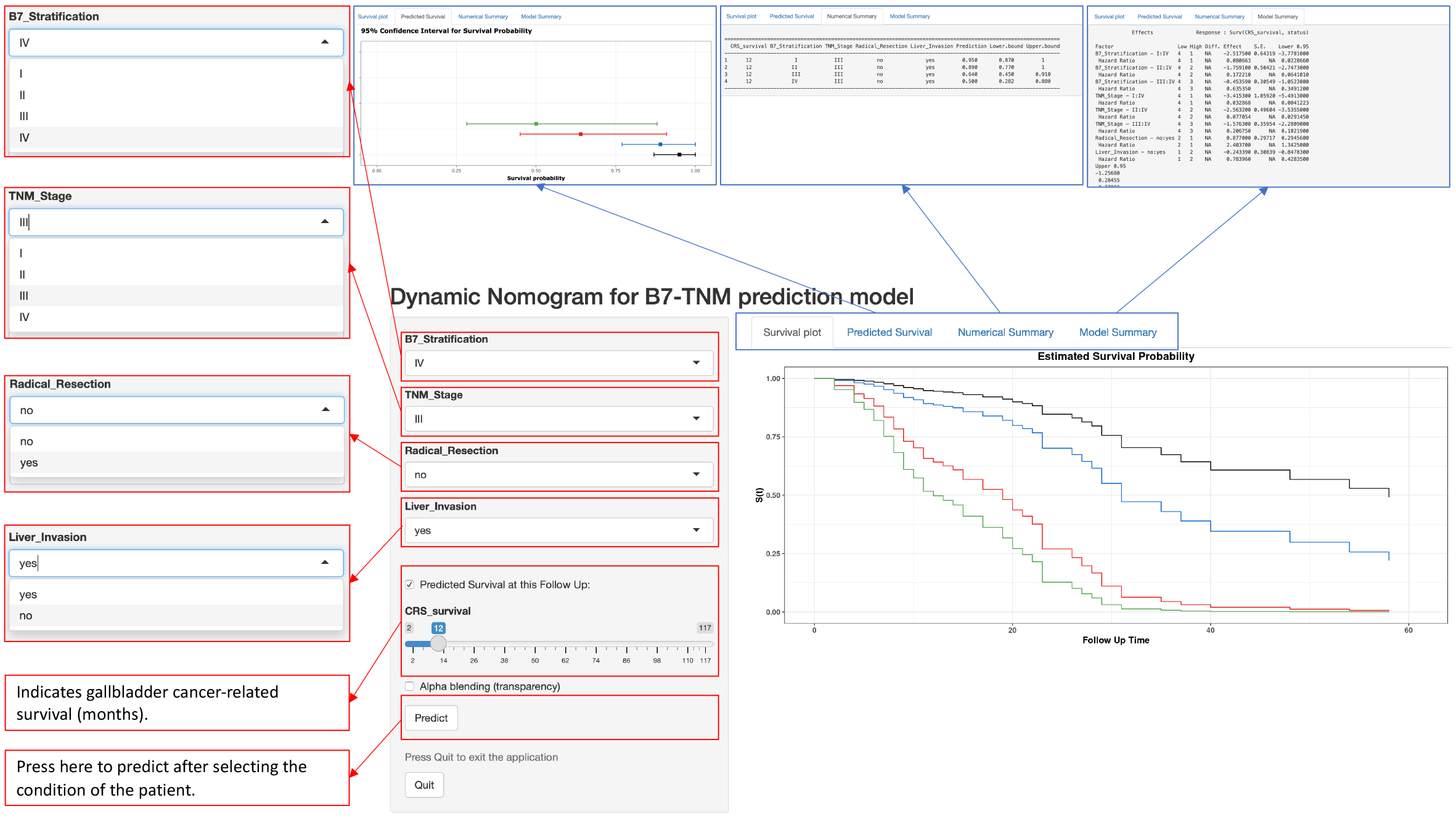

Supplement: Supplementary Figure 4 — Instructions for the dynamic nomogram of the B7-TNM prediction model on the webpage, Linkage to https://dynnomo-for-gallbladder-cancer.shinyapps.io/DynNomapp-B7_TNM_model/. [file Image_4.tif]

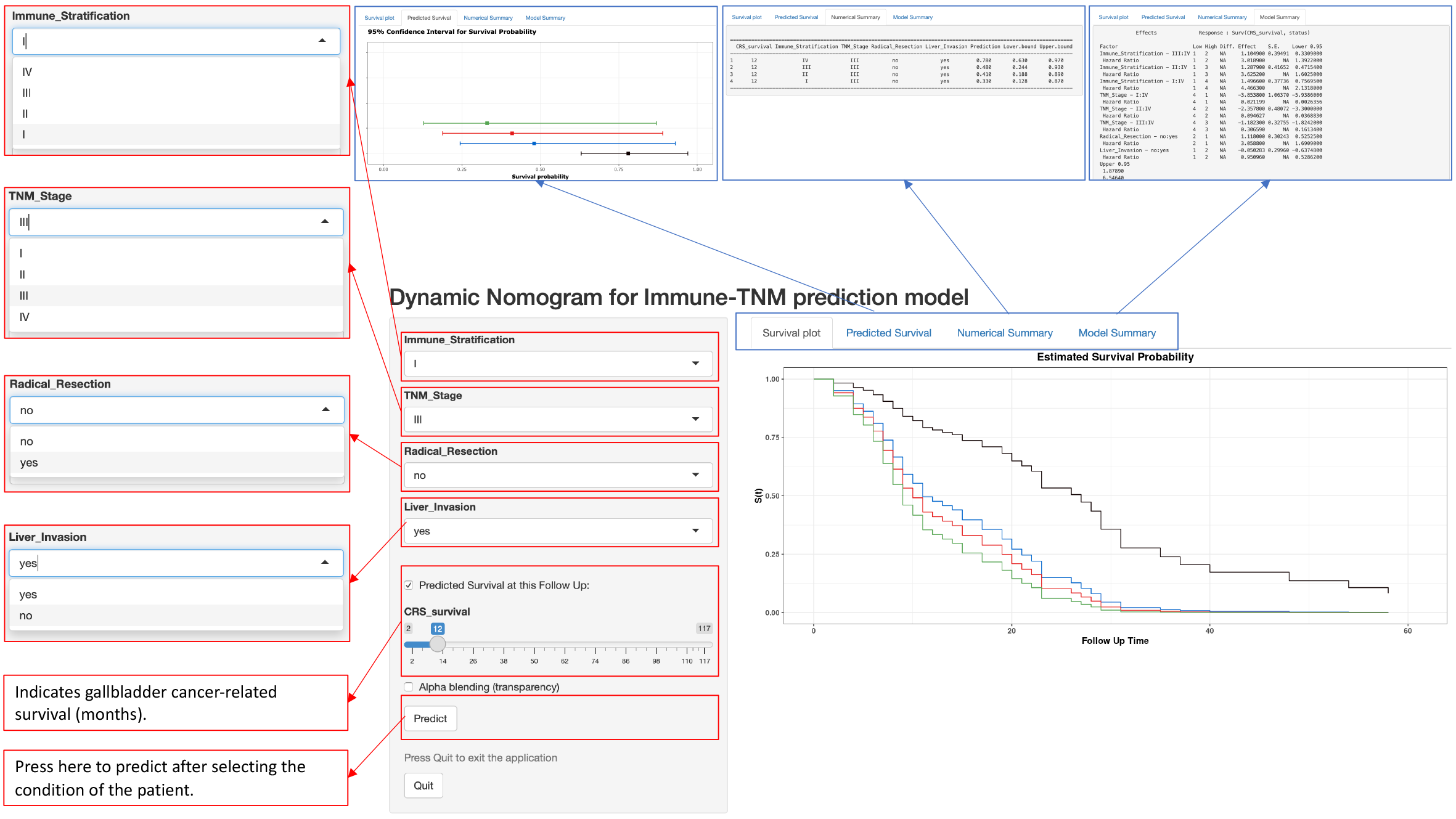

Supplement: Supplementary Figure 5 — Instructions for the dynamic nomogram of the Immune-TNM prediction model on the webpage, Linkage to https://dynnomo-for-gallbladder-cancer.shinyapps.io/DynNomapp-Immune_TNM_model/. [file Image_5.tif]
