# Supplementary material for: Novel immune scoring dynamic nomograms based on B7-H3, B7-H4, and HHLA2: Potential prediction in survival and immunotherapeutic efficacy for gallbladder cancer
Source: Front Immunol. 2022 Sep 8;13:984172. doi: 10.3389/fimmu.2022.984172 (PMC9493478; doi:10.3389/fimmu.2022.984172)
Supplement: Supplementary file 6 [file Table_1.docx]

| **Supplemental Table 1** Clinicopathological characteristics of gallbladder cancer patients | | | | | | |  |
| --- | --- | --- | --- | --- | --- | --- | --- |
|  |  | **Training group** | |  | **Testing group** | |  |
| **Variables** | | **No.** | **%** |  | **No.** | **%** | **P** |
| **Sex** | |  |  |  |  |  |  |
|  | Male | 36 | 37.89 |  | 40 | 38.83 | 0.892 |
|  | Female | 59 | 62.11 |  | 63 | 61.17 |  |
| **Age, mean±SD (range, year)** | | 62.33±10.65 | (30, 89) |  | 61.74±9.42 | (40, 87) | 0.688 |
| **Differentiation** | |  |  |  |  |  |  |
|  | Well | 30 | 31.58 |  | 30 | 29.13 | 0.940 |
|  | Moderate | 32 | 33.68 |  | 35 | 33.98 |  |
|  | Poor | 26 | 27.37 |  | 31 | 30.10 |  |
|  | Undifferentiated | 7 | 7.37 |  | 6 | 5.83 |  |
| **Histological type** | |  |  |  |  |  |  |
|  | Adenocarcinoma NOS | 79 | 83.16 |  | 74 | 71.84 | 0.336 |
|  | Papillary adenocarcinoma | 5 | 5.26 |  | 13 | 12.62 |  |
|  | Mucinous adenocarcinoma | 3 | 3.16 |  | 3 | 2.91 |  |
|  | Squamous cell carcinoma | 1 | 1.05 |  | 4 | 3.88 |  |
|  | Adenosquamous carcinoma | 4 | 4.21 |  | 4 | 3.88 |  |
|  | others | 3 | 3.16 |  | 5 | 4.85 |  |
| **Nevin stage** | |  |  |  |  |  |  |
|  | I | 4 | 4.21 |  | 8 | 7.77 | 0.477 |
|  | II | 15 | 15.79 |  | 10 | 9.71 |  |
|  | III | 16 | 16.84 |  | 19 | 18.45 |  |
|  | IV | 11 | 11.58 |  | 8 | 7.77 |  |
|  | V | 49 | 51.58 |  | 58 | 56.31 |  |
| **TNM stage** | |  |  |  |  |  |  |
|  | I | 11 | 11.58 |  | 10 | 9.71 | 0.970 |
|  | II | 22 | 23.16 |  | 24 | 23.30 |  |
|  | III | 21 | 22.11 |  | 22 | 21.36 |  |
|  | IV | 41 | 43.16 |  | 47 | 45.63 |  |
| **T stage** | |  |  |  |  |  |  |
|  | T1 | 11 | 11.58 |  | 10 | 9.71 | 0.970 |
|  | T2 | 31 | 32.63 |  | 34 | 33.01 |  |
|  | T3 | 30 | 31.58 |  | 32 | 31.07 |  |
|  | T4 | 23 | 24.21 |  | 27 | 26.21 |  |
| **N stage** | |  |  |  |  |  |  |
|  | N0 | 47 | 49.47 |  | 49 | 47.57 | 0.489 |
|  | N1 | 41 | 43.16 |  | 50 | 48.54 |  |
|  | N2 | 7 | 7.37 |  | 4 | 3.88 |  |
| **M stage** | |  |  |  |  |  |  |
|  | M0 | 57 | 60.00 |  | 65 | 63.11 | 0.653 |
|  | M1 | 38 | 40.00 |  | 38 | 36.89 |  |
| **Size, mean±SD (range, cm)** | | 3.9±2.1 | (1.0, 10.0) |  | 4.0±2.7 | (0.7, 18) | 0.709 |
| **Tumor site** | |  |  |  |  |  |  |
|  | Fundus, body | 61 | 64.21 |  | 63 | 61.17 | 0.658 |
|  | Neck，cystic duct | 34 | 35.79 |  | 40 | 38.83 |  |
| **Liver invasion** | |  |  |  |  |  |  |
|  | Yes | 32 | 33.68 |  | 41 | 39.81 | 0.372 |
|  | No | 63 | 66.32 |  | 62 | 60.19 |  |
| **Biliary tract invasion** | |  |  |  |  |  |  |
|  | Yes | 31 | 32.63 |  | 37 | 35.92 | 0.626 |
|  | No | 64 | 67.37 |  | 66 | 64.08 |  |
| **Operation** | |  |  |  |  |  |  |
|  | Radical resection | 67 | 70.53 |  | 69 | 66.99 | 0.592 |
|  | Palliative resection | 28 | 29.47 |  | 34 | 33.01 |  |
| **Complete resection** | |  |  |  |  |  |  |
|  | Yes | 80 | 84.21 |  | 86 | 83.50 | 0.891 |
|  | No | 15 | 15.79 |  | 17 | 16.50 |  |
| **Adjuvant chemotherapy** | |  |  |  |  |  |  |
|  | Yes | 7 | 7.37 |  | 10 | 9.71 | 0.557 |
|  | No | 88 | 92.63 |  | 93 | 90.29 |  |
| **Follow-up, mean±SD (range, year)** | | 32±30 | (0, 117) |  | 31±30 | (0, 115) | 0.783 |
| **NOS, not otherwise specified; SD, standard deviation** | | | | | | |  |
