# Supplementary material for: Novel immune scoring dynamic nomograms based on B7-H3, B7-H4, and HHLA2: Potential prediction in survival and immunotherapeutic efficacy for gallbladder cancer
Source: Front Immunol. 2022 Sep 8;13:984172. doi: 10.3389/fimmu.2022.984172 (PMC9493478; doi:10.3389/fimmu.2022.984172)
Supplement: Supplementary file 7 [file Table_2.docx]

| **Supplemental Table 2** The association of B7-H3, B7-H4, and HHLA2 expression with clinicopathological characteristics in both groups | | | | | | | | | | | | | | | | | | | | | | | | | | | | |
| --- | --- | --- | --- | --- | --- | --- | --- | --- | --- | --- | --- | --- | --- | --- | --- | --- | --- | --- | --- | --- | --- | --- | --- | --- | --- | --- | --- | --- |
| **Variables** | | **Training group** | | | | | | | | | | | | |  | **Testing group** | | | | | | | | | | | | |
|  |  |  |  | **B7-H3** | | |  | **B7-H4** | | |  | **HHLA2** | | |  |  |  | **B7-H3** | | |  | **B7-H4** | | |  | **HHLA2** | | |
|  |  | **No.** | **%** | **-** | **+** | **p** |  | **-** | **+** | **p** |  | **-** | **+** | **p** |  | **No.** | **%** | **-** | **+** | **p** |  | **-** | **+** | **p** |  | **-** | **+** | **p** |
| **Differentiation** | |  |  |  |  |  |  |  |  |  |  |  |  |  |  |  |  |  |  |  |  |  |  |  |  |  |  |  |
|  | Poor, undifferentiation | 33 | 34.74 | 8 | 25 | 0.420 |  | 13 | 20 | 0.594 |  | 15 | 18 | 0.904 |  | 37 | 35.92 | 10 | 27 | 0.419 |  | 8 | 29 | 0.091 |  | 17 | 20 | 0.345 |
|  | Well, Moderate | 62 | 65.26 | 20 | 42 |  |  | 28 | 34 |  |  | 29 | 33 |  |  | 66 | 64.08 | 23 | 43 |  |  | 25 | 41 |  |  | 24 | 42 |  |
| **Nevin stage** | |  |  |  |  |  |  |  |  |  |  |  |  |  |  |  |  |  |  |  |  |  |  |  |  |  |  |  |
|  | IV, V | 60 | 63.16 | 7 | 53 | <0.001 |  | 18 | 42 | 0.001 |  | 22 | 38 | 0.013 |  | 66 | 64.08 | 15 | 51 | 0.007 |  | 14 | 52 | 0.001 |  | 21 | 45 | 0.027 |
|  | I, II, III | 35 | 36.84 | 21 | 14 |  |  | 23 | 12 |  |  | 22 | 13 |  |  | 37 | 35.92 | 18 | 19 |  |  | 19 | 18 |  |  | 20 | 17 |  |
| **TNM stage** | |  |  |  |  |  |  |  |  |  |  |  |  |  |  |  |  |  |  |  |  |  |  |  |  |  |  |  |
|  | III, IV | 62 | 65.26 | 8 | 54 | <0.001 |  | 20 | 42 | 0.003 |  | 23 | 39 | 0.013 |  | 68 | 66.02 | 16 | 52 | 0.010 |  | 14 | 54 | <0.001 |  | 21 | 47 | 0.010 |
|  | I, II | 33 | 34.74 | 20 | 13 |  |  | 21 | 12 |  |  | 21 | 12 |  |  | 35 | 33.98 | 17 | 18 |  |  | 19 | 16 |  |  | 20 | 15 |  |
| **T stage** | T3, T4 | 53 | 55.79 | 6 | 47 | <0.001 |  | 13 | 40 | <0.001 |  | 18 | 35 | 0.006 |  | 59 | 57.28 | 14 | 45 | 0.037 |  | 12 | 47 | 0.003 |  | 20 | 39 | 0.159 |
|  | T1, T2 | 42 | 44.21 | 22 | 20 |  |  | 28 | 14 |  |  | 26 | 16 |  |  | 44 | 42.72 | 19 | 25 |  |  | 21 | 23 |  |  | 21 | 23 |  |
| **N stage** | N1, N2 | 48 | 50.53 | 5 | 43 | <0.001 |  | 15 | 33 | 0.018 |  | 16 | 32 | 0.010 |  | 54 | 52.43 | 14 | 40 | 0.166 |  | 11 | 43 | 0.007 |  | 17 | 37 | 0.071 |
|  | N0 | 47 | 49.47 | 23 | 24 |  |  | 26 | 21 |  |  | 28 | 19 |  |  | 49 | 47.57 | 19 | 30 |  |  | 22 | 27 |  |  | 24 | 25 |  |
| **M stage** | M1 | 38 | 40.00 | 3 | 35 | <0.001 |  | 10 | 28 | 0.006 |  | 12 | 26 | 0.018 |  | 38 | 36.89 | 6 | 32 | 0.007 |  | 2 | 36 | <0.001 |  | 8 | 30 | 0.003 |
|  | M0 | 57 | 60.00 | 25 | 32 |  |  | 31 | 26 |  |  | 32 | 25 |  |  | 65 | 63.11 | 27 | 38 |  |  | 31 | 34 |  |  | 33 | 32 |  |
| **B7-H4** | + | 54 | 56.84 | 10 | 44 | 0.007 |  | - | | |  | - | | |  | 70 | 67.96 | 15 | 55 | 0.001 |  | - |  |  |  | - | | |
|  | - | 41 | 43.16 | 18 | 23 |  |  |  |  |  |  |  |  |  |  | 33 | 32.04 | 18 | 15 |  |  |  |  |  |  |  |  |  |
| **HHLA2** | + | 51 | 53.68 | 10 | 41 | 0.023 |  | 15 | 36 | 0.003 |  | - | | |  | 62 | 60.19 | 12 | 50 | 0.001 |  | 12 | 50 | 0.001 |  | - | | |
|  | - | 44 | 46.32 | 18 | 26 |  |  | 26 | 18 |  |  |  |  |  |  | 41 | 39.81 | 21 | 20 |  |  | 21 | 20 |  |  |  |  |  |
| +, high expression; -, low expression; p value ＜0.05 is statistically significant. | | | | | | | | | | | | | | | | | | | | | | | | | | | | |
