# Supplementary material for: Novel immune scoring dynamic nomograms based on B7-H3, B7-H4, and HHLA2: Potential prediction in survival and immunotherapeutic efficacy for gallbladder cancer
Source: Front Immunol. 2022 Sep 8;13:984172. doi: 10.3389/fimmu.2022.984172 (PMC9493478; doi:10.3389/fimmu.2022.984172)
Supplement: Supplementary file 8 [file Table_3.docx]

| **Supplemental Table 3** Univariate analysis of clinicopathological parameters associated with OS and CRS | | | | | | | | | | | | | | | | |
| --- | --- | --- | --- | --- | --- | --- | --- | --- | --- | --- | --- | --- | --- | --- | --- | --- |
| **Variables** | | **Training group** | | | | | | |  | **Testing group** | | | | | | |
|  |  | **OS** | | |  | **CRS** | | |  | **OS** | | |  | **CRS** | | |
|  |  | **HR** | **95% CI** | **p** |  | **HR** | **95% CI** | **p** |  | **HR** | **95% CI** | **p** |  | **HR** | **95% CI** | **p** |
| **Age** (≥60 years/<60 years) | | 1.14 | (0.70 to 1.85) | 0.610 |  | 1.17 | (0.70 to 1.95) | 0.551 |  | 1.03 | (0.65 to 1.63) | 0.905 |  | 1.13 | (0.69 to 1.84) | 0.639 |
| **Differentiation** | |  |  |  |  |  |  |  |  |  |  |  |  |  |  |  |
|  | (Poor, undifferentiation/ | 1.87 | (1.13 to 3.08) | 0.014 |  | 1.95 | (1.16 to 3.28) | 0.012 |  | 2.19 | (1.37 to 3.50) | 0.001 |  | 1.95 | (1.18 to 3.21) | 0.009 |
|  | Well, and Moderate) |  |  |  |  |  |  |  |  |  |  |  |  |  |  |  |
| **Nevin stage** (IV, V/I, II, III) | | 6.86 | (3.59 to 13.14) | <0.001 |  | 6.88 | (3.43 tp 13.80) | <0.001 |  | 5.51 | (3.02 to 10.07) | <0.001 |  | 5.62 | (2.90 to 10.90) | <0.001 |
| **TNM stage** (III, IV/I, II) | | 7.96 | (3.96 to 16.01) | <0.001 |  | 8.23 | (3.84 to 17.64) | <0.001 |  | 6.86 | (3.45 to 13.66) | <0.001 |  | 7.41 | (3.46 to 15.84) | <0.001 |
| **T stage** (T3, T4/T1, T2) | | 8.21 | (4.46 to 15.12) | <0.001 |  | 7.86 | (4.16 to 14.87) | <0.001 |  | 6.12 | (3.43 to 10.94) | <0.001 |  | 5.56 | (3.05 to 10.14) | <0.001 |
| **N stage** (N1, N2/N0) | | 4.68 | (2.70 to 8.09) | <0.001 |  | 4.71 | (2.65 to 8.37) | <0.001 |  | 3.57 | (2.16 to 5.89) | <0.001 |  | 3.54 | (2.07 to 6.06) | <0.001 |
| **M stage** (M1/M0) | | 9.06 | (5.13 to 16.03) | <0.001 |  | 8.14 | (4.58 to 14.47) | <0.001 |  | 11.75 | (6.13 to 22.52) | <0.001 |  | 10.16 | (5.22 to 19.76) | <0.001 |
| **B7-H3** (+/-) | | 6.65 | (3.10 to 14.28) | <0.001 |  | 6.04 | (2.58 to 14.14) | <0.001 |  | 3.98 | (2.18 to 7.27) | <0.001 |  | 4.13 | (2.18 to 7.83) | <0.001 |
| **B7-H4** (+/-) | | 3.12 | (1.81 to 5.39) | <0.001 |  | 3.24 | (1.82 to 5.76) | <0.001 |  | 6.13 | (3.09 to 12.15) | <0.001 |  | 5.78 | (2.83 to 11.82) | <0.001 |
| **HHLA2** (+/-) | | 3.21 | (1.88 to 5.47) | <0.001 |  | 3.39 | (1.96 to 5.86) | <0.001 |  | 2.76 | (1.64 to 4.64) | <0.001 |  | 2.59 | (1.48 to 4.52) | 0.001 |
| **CD8** (high/low) | | 0.62 | (0.38 to 1.01) | 0.056 |  | 0.56 | (0.34 to 0.93) | 0.026 |  | 0.59 | (0.37 to 0.93) | 0.023 |  | 0.55 | (0.34 to 0.90) | 0.018 |
| **Size** (≥3.5 cm/<3.5 cm) | | 2.08 | (1.26 to 3.45) | 0.004 |  | 2.03 | (1.20 to 3.44) | 0.009 |  | 2.63 | (1.62 to 4.27) | <0.001 |  | 2.76 | (1.64 to 4.64) | <0.001 |
| **Tumor site** | |  |  |  |  |  |  |  |  |  |  |  |  |  |  |  |
|  | (Neck, cystic duct/ | 2.43 | (1.47 to 4.02) | 0.001 |  | 2.46 | (1.46 to 4.13) | 0.001 |  | 3.32 | (2.03 to 5.41) | <0.001 |  | 2.93 | (1.76 to 4.90) | <0.001 |
|  | Fundus, body) |  |  |  |  |  |  |  |  |  |  |  |  |  |  |  |
| **Liver invasion** (Yes/No) | | 3.15 | (1.88 to 5.23) | <0.001 |  | 2.98 | (1.75 to 5.07) | <0.001 |  | 4.01 | (2.45 to 6.57) | <0.001 |  | 3.66 | (2.19 to 6.12) | <0.001 |
| **Biliary tract invasion** (Yes/No) | | 3.18 | (1.90 to 5.30) | <0.001 |  | 3.23 | (1.91 to 5.46) | <0.001 |  | 4.24 | (2.55 to 7.05) | <0.001 |  | 3.76 | (2.21 to 6.41) | <0.001 |
| **Operation** | |  |  |  |  |  |  |  |  |  |  |  |  |  |  |  |
|  | (Palliative resection/ | 4.10 | (2.44 to 6.91) | <0.001 |  | 4.86 | (2.74 to 8.63) | <0.001 |  | 3.74 | (2.28 to 6.13) | <0.001 |  | 4.01 | (2.37 to 6.78) | <0.001 |
|  | Radical resection) |  |  |  |  |  |  |  |  |  |  |  |  |  |  |  |
| **Complete resection** (No/Yes) | | 2.69 | (1.49 to 4.88) | 0.001 |  | 2.81 | (1.54 to 5.11) | 0.001 |  | 2.39 | (1.34 to 4.26) | 0.003 |  | 2.64 | (1.44 to 4.82) | 0.002 |
| **Adjuvant chemotherapy** (No/Yes) | | 2.19 | (0.69 to 6.98) | 0.186 |  | 2.63 | (0.64 to 10.80) | 0.178 |  | 1.28 | (0.61 to 2.68) | 0.521 |  | 1.36 | (0.644 to 2.87) | 0.421 |
| +, high expression; -, low expression; HR, hazard ratio; CI, confident interval; p value ＜0.05 is statistically significant; OS, overall survival; CRS, cancer-related survival. | | | | | | | | | | | | | | | | |
