# Supplementary material for: Novel immune scoring dynamic nomograms based on B7-H3, B7-H4, and HHLA2: Potential prediction in survival and immunotherapeutic efficacy for gallbladder cancer
Source: Front Immunol. 2022 Sep 8;13:984172. doi: 10.3389/fimmu.2022.984172 (PMC9493478; doi:10.3389/fimmu.2022.984172)
Supplement: Supplementary file 9 [file Table_4.docx]

| **Supplemental Table 4** Multivariate analysis of clinicopathological parameters associated with OS and CRS based on B7-H3, B7-H4, and HHLA2 | | | | | | | | | | | | | | | | |
| --- | --- | --- | --- | --- | --- | --- | --- | --- | --- | --- | --- | --- | --- | --- | --- | --- |
| **Variables** | | **Training group** | | | | | | |  | **Testing group** | | | | | | |
|  |  | **OS** | | |  | **CRS** | | |  | **OS** | | |  | **CRS** | | |
|  |  | **HR** | **95% CI** | **p** |  | **HR** | **95% CI** | **p** |  | **HR** | **95% CI** | **p** |  | **HR** | **95% CI** | **p** |
| **Differentiation** | |  |  |  |  |  |  |  |  |  |  |  |  |  |  |  |
|  | (Poor, and undifferentiation/ | 1.61 | (0.88 to 2.97) | 0.125 |  | 1.57 | (0.82 to 3.00) | 0.175 |  | 1.66 | (0.92 to 3.01) | 0.093 |  | 1.66 | (0.88 to 3.16) | 0.119 |
|  | Well, and Moderate) |  |  |  |  |  |  |  |  |  |  |  |  |  |  |  |
| **Nevin stage** (IV, V/I, II, III) | | 22.41 | (2.77 to 181.03) | 0.004 |  | 16.55 | (1.91 to 143.28) | 0.011 |  | 2.34 | (0.66 to 8.31) | 0.189 |  | 1.80 | (0.48 to 6.68) | 0.38 |
| **TNM stage** (III, IV/I, II) | | 20.53 | (2.86 to 147.34) | 0.003 |  | 23.47 | (3.03 to 181.72) | 0.003 |  | 1.31 | (0.30 to 5.64) | 0.722 |  | 2.50 | (0.57 to 11.01) | 0.226 |
| **T stage** (T3, T4/T1, T2) | | 9.86 | (2.39 to 40.70) | 0.002 |  | 7.61 | (1.75 to 33.05) | 0.007 |  | 1.01 | (0.31 to 3.31) | 0.993 |  | 1.47 | (0.44 to 4.93) | 0.53 |
| **N stage** (N1, N2/N0) | | 2.11 | (0.80 to 5.57) | 0.134 |  | 2.91 | (1.03 to 8.22) | 0.043 |  | 1.49 | (0.79 to 2.80) | 0.214 |  | 1.41 | (0.75 to 2.67) | 0.292 |
| **M stage** (M1/M0) | | 7.82 | (3.55 to 17.25) | <0.001 |  | 6.07 | (2.74 to 13.43) | <0.001 |  | 3.79 | (1.79 to 8.01) | <0.001 |  | 3.15 | (1.44 to 6.87) | 0.004 |
| **B7-H3** (+/-) | | 6.18 | (2.40 to 15.91) | <0.001 |  | 3.67 | (1.29 to 10.40) | 0.015 |  | 3.47 | (1.69 to 7.12) | 0.001 |  | 4.61 | (2.1 to 10.12) | <0.001 |
| **B7-H4** (+/-) | | 1.34 | (0.68 to 2.65) | 0.403 |  | 1.34 | (0.63 to 2.85) | 0.441 |  | 2.58 | (1.19 to 5.59) | 0.016 |  | 2.29 | (0.99 to 5.31) | 0.054 |
| **HHLA2** (+/-) | | 3.67 | (1.83 to 7.37) | <0.001 |  | 2.64 | (1.30 to 5.36) | 0.007 |  | 1.24 | (0.68 to 2.26) | 0.483 |  | 1.11 | (0.57 to 2.19 | 0.755 |
| **CD8** (high/low) | | 0.8 | (0.41 to 1.56) | 0.515 |  | 0.53 | (0.25 to 1.09) | 0.085 |  | 0.75 | (0.46 to 1.25) | 0.271 |  | 0.71 | (0.42 to 1.23) | 0.221 |
| **Size** (≥3.5 cm/<3.5 cm) | | 1.36 | (0.70 to 2.65) | 0.368 |  | 1.54 | (0.74 to 3.17) | 0.247 |  | 1.29 | (0.66 to 2.53) | 0.454 |  | 1.58 | (0.74 to 3.38) | 0.234 |
| **Tumor site** | |  |  |  |  |  |  |  |  |  |  |  |  |  |  |  |
|  | (Neck，cystic duct/ | 2.01 | (0.85 to 4.72) | 0.111 |  | 1.73 | (0.71 to 4.20) | 0.226 |  | 1.01 | (0.46 to 2.25) | 0.976 |  | 1.07 | (0.46 to 2.47) | 0.873 |
|  | Fundus, body) |  |  |  |  |  |  |  |  |  |  |  |  |  |  |  |
| **Liver invasion** (Yes/No) | | 2.44 | (1.16 to 5.13) | 0.019 |  | 2.53 | (1.11 to 5.75) | 0.026 |  | 1.69 | (0.86 to 3.32) | 0.128 |  | 2.02 | (0.98 to 4.15) | 0.056 |
| **Biliary tract invasion** (Yes/No) | | 1.89 | (0.82 to 4.38) | 0.136 |  | 1.85 | (0.76 to 4.51) | 0.173 |  | 1.19 | (0.54 to 2.64) | 0.66 |  | 1.01 | (0.45 to 2.27) | 0.99 |
| **Operation** | |  |  |  |  |  |  |  |  |  |  |  |  |  |  |  |
|  | (Palliative resection/ | 3.83 | (1.80 to 8.18) | 0.001 |  | 5.35 | (2.13 to 13.44) | <0.001 |  | 3.38 | (1.61 to 7.12) | 0.001 |  | 3.46 | (1.48 to 8.06 | 0.004 |
|  | Radical resection) |  |  |  |  |  |  |  |  |  |  |  |  |  |  |  |
| **Complete resection** (No/Yes) | | 1.768 | (0.87 to 3.61) | 0.118 |  | 1.93 | (0.92 to 4.05) | 0.082 |  | 1.74 | (0.82 to 3.68) | 0.15 |  | 2.49 | (1.09 to 5.72) | 0.031 |
| +, high expression; -, low expression; HR, hazard ratio; CI, confident interval; p value ＜0.05 is statistically significant; OS, overall survival; CRS, cancer-related survival. | | | | | | | | | | | | | | | | |
