# Supplementary material for: Novel immune scoring dynamic nomograms based on B7-H3, B7-H4, and HHLA2: Potential prediction in survival and immunotherapeutic efficacy for gallbladder cancer
Source: Front Immunol. 2022 Sep 8;13:984172. doi: 10.3389/fimmu.2022.984172 (PMC9493478; doi:10.3389/fimmu.2022.984172)
Supplement: Supplementary file 10 [file Table_5.docx]

| **Supplemental Table 5** The association of B7 stratification with clinicopathological characteristics in both groups | | | | | | | | | | | | |
| --- | --- | --- | --- | --- | --- | --- | --- | --- | --- | --- | --- | --- |
| **Variables** | | **Training group** | | | | |  | **Testing group** | | | | |
|  |  |  |  | **B7 stratification** | | |  |  |  | **B7 stratification** | | |
|  |  | **No.** | **%** | **low grade** | **high grade** | **p** |  | **No.** | **%** | **low grade** | **high grade** | **p** |
| **Differentiation** | |  |  |  |  |  |  |  |  |  |  |  |
|  | Poor, undifferentiation | 33 | 34.737 | 21 | 12 | 0.648 |  | 37 | 35.92 | 21 | 16 | 0.532 |
|  | Well, Moderate | 62 | 65.263 | 43 | 19 |  |  | 66 | 64.08 | 42 | 24 |  |
| **Nevin stage** | |  |  |  |  |  |  |  |  |  |  |  |
|  | IV, V | 60 | 63.158 | 31 | 29 | <0.001 |  | 66 | 64.08 | 34 | 32 | 0.011 |
|  | I, II, III | 35 | 36.842 | 33 | 2 |  |  | 37 | 35.92 | 29 | 8 |  |
| **TNM stage** | |  |  |  |  |  |  |  |  |  |  |  |
|  | III, IV | 62 | 65.263 | 33 | 29 | <0.001 |  | 68 | 66.02 | 35 | 33 | 0.006 |
|  | I, II | 33 | 34.737 | 31 | 2 |  |  | 35 | 33.98 | 28 | 7 |  |
| **T stage** | |  |  |  |  |  |  |  |  |  |  |  |
|  | T3, T4 | 53 | 55.789 | 26 | 27 | <0.001 |  | 59 | 57.28 | 31 | 28 | 0.043 |
|  | T1, T2 | 42 | 44.211 | 38 | 4 |  |  | 44 | 42.72 | 32 | 12 |  |
| **N stage** | |  |  |  |  |  |  |  |  |  |  |  |
|  | N1, N2 | 48 | 50.526 | 26 | 22 | 0.008 |  | 54 | 52.43 | 31 | 23 | 0.427 |
|  | N0 | 47 | 49.474 | 38 | 9 |  |  | 49 | 47.57 | 32 | 17 |  |
| **M stage** | |  |  |  |  |  |  |  |  |  |  |  |
|  | M1 | 38 | 40 | 17 | 21 | <0.001 |  | 38 | 36.89 | 14 | 24 | <0.001 |
|  | M0 | 57 | 60 | 47 | 10 |  |  | 65 | 63.11 | 49 | 16 |  |
| p value ＜0.05 is statistically significant. | | | | | | | | | | | | |
