# Supplementary material for: Novel immune scoring dynamic nomograms based on B7-H3, B7-H4, and HHLA2: Potential prediction in survival and immunotherapeutic efficacy for gallbladder cancer
Source: Front Immunol. 2022 Sep 8;13:984172. doi: 10.3389/fimmu.2022.984172 (PMC9493478; doi:10.3389/fimmu.2022.984172)
Supplement: Supplementary file 11 [file Table_6.docx]

| **Supplemental Table 6** A List of C-index for prediction models of cancer-related survival (CRS) for gallbladder cancer | | | | | |
| --- | --- | --- | --- | --- | --- |
| **Prediction models** | **Associated risk factors** | **Groups** | **Novel model** | **Staging system** | **p** |
| **B7-TNM model** | B7 stratification, **TNM stage,** radical resection, liver invasion | Training | 0.96 (0.92, 0.99) | *0.88 (0.82, 0.95) | 0.058 |
|  |  | Testing | 0.94 (0.90, 0.98) | *0.90 (0.84, 0.96) | 0.129 |
| **Immune-TNM model** | Immune stratification, **TNM stage**, radical resection, liver invasion | Training | 0.94 (0.89, 0.98) | *0.88 (0.82, 0.95) | 0.062 |
|  |  | Testing | 0.95 (0.90, 0.99) | *0.90 (0.84, 0.96) | 0.137 |
| **B7-Nevin model** | B7 stratification, **Nevin stage,** radical resection, liver invasion | Training | 0.92 (0.86, 0.97) | #0.82 (0.73, 0.91) | 0.066 |
|  |  | Testing | 0.95 (0.91, 0.99) | #0.86 (0.79, 0.94) | 0.061 |
| **Immune-Nevin model** | Immune stratification, **Nevin stage,** radical resection, liver invasion | Training | 0.93 (0.87, 0.98) | #0.82 (0.73, 0.91) | 0.066 |
|  |  | Testing | 0.91 (0.85, 0.97) | #0.86 (0.79, 0.94) | 0.096 |
| Data is listed as C-index with 95% confidence interval (CI); *, indicates C-index of TNM staging system, #, indicates C-index of Nevin staging system | | | | | |
