# Supplementary material for: Novel immune scoring dynamic nomograms based on B7-H3, B7-H4, and HHLA2: Potential prediction in survival and immunotherapeutic efficacy for gallbladder cancer
Source: Front Immunol. 2022 Sep 8;13:984172. doi: 10.3389/fimmu.2022.984172 (PMC9493478; doi:10.3389/fimmu.2022.984172)
Supplement: Supplementary file 12 [file Table_7.docx]

| **Supplemental Table 7** Multivariate regression model parameters for Dynamic nomograms. | | |
| --- | --- | --- |
| **Covariate** | **Beta Coefficient** | **p** |
| **B7-TNM prediction model** | | |
| **B7 stratification** |  |  |
| I | 1.0000 | - |
| II | 0.7584 | 0.2842 |
| III | 2.0639 | 0.0014 |
| IV | 2.5175 | <0.0001 |
| **TNM stage** |  |  |
| I | 1.0000 | - |
| II | 0.8520 | 0.4290 |
| III | 1.8390 | 0.0810 |
| IV | 3.4153 | 0.0013 |
| **Radical Resection (yes)** | -0.8770 | 0.0032 |
| **Liver invasion (no)** | -0.2434 | 0.4300 |
|  | | |
| **Immune-TNM prediction model** | | |
| **Immune stratification** | |  |
| IV | 1.0000 | - |
| III | 1.1049 | 0.0051 |
| II | 1.2879 | 0.0020 |
| I | 1.4966 | <0.0001 |
| **TNM stage** |  |  |
| I | 1.0000 | - |
| II | 1.4960 | 0.1644 |
| III | 2.6710 | 0.0126 |
| IV | 3.8538 | 0.0003 |
| **Radical Resection (yes)** | -1.1180 | 0.0002 |
| **Liver invasion (no)** | -0.0503 | 0.8667 |
